# Two-dimensional materials in functional three-dimensional architectures with applications in photodetection and imaging

**DOI:** 10.1038/s41467-018-03870-0

**Published:** 2018-04-12

**Authors:** Wonho Lee, Yuan Liu, Yongjun Lee, Bhupendra K. Sharma, Sachin M. Shinde, Seong Dae Kim, Kewang Nan, Zheng Yan, Mengdi Han, Yonggang Huang, Yihui Zhang, Jong-Hyun Ahn, John A. Rogers

**Affiliations:** 1 School of Electrical and Electronic Engineering, Yonsei University, 50 Yonsei-ro, Seoul, 03722 Republic of Korea; 2 Department of Engineering Mechanics, Center for Mechanics and Materials and Center for Flexible Electronics Technology, AML, Tsinghua University, 100084 Beijing, China; 3 Departments of Materials Science and Engineering, Biomedical Engineering, Chemistry, Mechanical Engineering, Electrical Engineering and Computer Science, Center for Bio-Integrated Electronics, Simpson Querrey Institute for Nano/Biotechnology, Northwestern University, Evanston, IL 60208 USA; 4 Departments of Civil and Environmental Engineering, Mechanical Engineering, and Materials Science and Engineering, Northwestern University, Evanston, IL 60208 USA

## Abstract

Efficient and highly functional three-dimensional systems that are ubiquitous in biology suggest that similar design architectures could be useful in electronic and optoelectronic technologies, extending their levels of functionality beyond those achievable with traditional, planar two-dimensional platforms. Complex three-dimensional structures inspired by origami, kirigami have promise as routes for two-dimensional to three-dimensional transformation, but current examples lack the necessary combination of functional materials, mechanics designs, system-level architectures, and integration capabilities for practical devices with unique operational features. Here, we show that two-dimensional semiconductor/semi-metal materials can play critical roles in this context, through demonstrations of complex, mechanically assembled three-dimensional systems for light-imaging capabilities that can encompass measurements of the direction, intensity and angular divergence properties of incident light. Specifically, the mechanics of graphene and MoS_2_, together with strategically configured supporting polymer films, can yield arrays of photodetectors in distinct, engineered three-dimensional geometries, including octagonal prisms, octagonal prismoids, and hemispherical domes.

## Abstract (web summary)

The strain tolerance and promising optoelectronic properties of 2D materials can be leveraged to design functional optical sensing devices. Here, the authors provide a demonstration of arrays of independently addressable photodetectors constructed from graphene and MoS_2_ engineered in 3D Kirigami geometries.

## Introduction

Electronic and optoelectronic materials deployed in complex, three-dimensional (3D) structures can offer qualitatively expanded levels of functionality compared to those in their corresponding two-dimensional (2D) planar counterparts. Many examples demonstrate clearly the value of 3D structures in achieving unique properties with simple constituent materials^1–5^. Progress in this area is often limited by the relatively small range of choices in controlled, reliable, reproducible strategies for producing 3D geometrical forms in advanced functional materials^6–11^. Among the most recently introduced methods is a scheme in which compressive buckling associated with a stretched elastomeric substrate guides the mechanical assembly of elaborate 3D mesostructures, some with designs reminiscent of those achieved in macroscale structures by origami/kirigami, with specified shapes and with sizes that can span several orders of magnitude in characteristic dimensions, down to the submicron regime in lateral features and to a few tens of nanometers in thickness^12–15^. These ideas leverage an intimate interplay between materials and microstructural mechanics, with diverse examples of use with silicon membranes, metallic electrodes, and polymer films in hundreds of different 3D geometries^13–16^. Full, quantitative modeling of the mechanics forms an essential aspect of design in all such cases; without such theoretical guidance, the buckling process itself can lead to cracking and/or defect formation in the constituent materials in ways that can deteriorate their properties. Physical toughness and materials structure geometries are, therefore, critically important in maximizing the diversity of realizable 3D structures.

Atomically thin, 2D materials have a well-established set of excellent mechanical properties, some of which, for graphene, are unmatched; these characteristics have direct and essential relevance in the context of 3D assembly^17–19^. In fact, recent work demonstrates that graphene can be built into common, kirigami-type structures and in mechanical metamaterials such as stretchable electrodes, springs, and hinges^20,21^. The assembly approaches in these cases, however, rely on externally imposed forces, with limited ability to address complex, 3D architectures and/or functional systems that form naturally in a parallel fashion with the requisite heterogeneous collection of patterned materials.

Here, we explore the use of 2D materials in functional, 3D systems formed via geometry transformation guided by compressive buckling, with a focus examples in constructs that provide 3D photodetection/imaging capabilities by use of light sensing elements that incorporate monolayer MoS_2_ and graphene, each of which offers an extraordinary combination of electrical, mechanical and optical properties relevant for present purposes. Specifically, graphene offers the best set of parameters for flexible, transparent conductors, with potential to replace indium–tin–oxide in flat panel displays and touch screens^22,23^. Additionally, MoS_2_ is of great interest for its excellent semiconducting properties at atomic thicknesses^24–26^. Owing to a direct band gap of 1.9 eV in monolayer MoS_2_, this material is well-suited for applications in the unique photodetecting systems^27–32^. Realizing 3D arrays of MoS_2_/graphene photodetectors involves first optimizing the parameters for assembly of origami-inspired 3D shapes (an octagonal prism, an octagonal prismoid, and a hemisphere) using finite-element analysis (FEA). The set of assembly parameters defined in this way serves as starting points for experimentally constructing the targeted 3D shapes, with integrated devices, by compressive buckling. Here, MoS_2_ and graphene serve as the channel and electrodes, respectively, in ultrathin semiconductor photoresistor supported by a layer of polymer. The resulting system forms spontaneously, and without external application of targeted forces, from a 2D planar geometry into a 3D configuration that allows tracking of both the direction and intensity of incident light. As an additional feature, the atomically thin MoS_2_ and graphene yield optically transparent devices, such that light passing through the device can be detected at two sensing locations (the entry and exit sites), thereby providing further information of relevance to divergence angle.

## Results

### Mechanically guided 3D transformation of 2D assembly: simulation and experiment

**Fig. 1:**
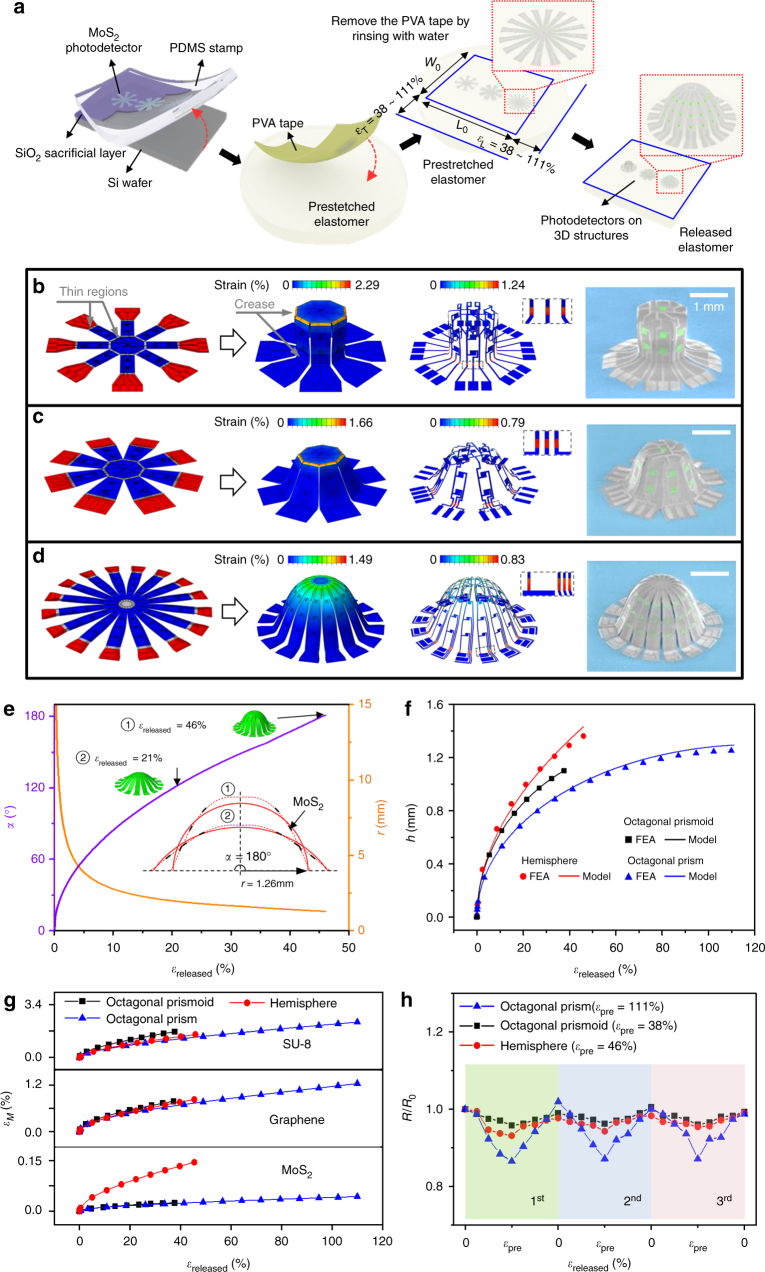
Assembly and mechanical analysis of 3D photodetector structures from 2D materials. **a** Schematic illustration of processes for fabricating the 3D systems. **b** FEA results describing the formation of arrays of photodetectors based on graphene and MoS_2_ in the form of an octagonal prism, and corresponding colorized SEM images of the final configuration including MoS_2_ (green), graphene (light gray), and SU-8 (gray). **c**, **d** Similar FEA results for the cases of an octagonal prismoid and a hemisphere. Colors represent the magnitude of the maximum principal strain. **e** Central angle and radius of a cross-section of the hemisphere in **d** vs. the released pre-strain. Here, the insets denote an intermediate state (*ε*_released_ = 21.4%) and the final state (*ε*_released_ = 46%) of pre-strain release, with the dashed and solid lines representing the profiles from FEA and fitting arcs, respectively. **f** FEA results and analytic predictions of the height of three photodetector structures in **b**–**d**. **g** Computational study of tensile strain applied to the SU-8, graphene, and MoS_2_ layers vs. pre-strain during the 3D assembly (squares, circles, and triangles denote the octagonal frustum, hemisphere, and octagonal prism, respectively). **h** Experimentally measured variation in the resistance in graphene during repeated buckling processes

Figure 1a provides a schematic illustration of the design and assembly process for a 3D photodetector system. Briefly, the complete device results from sequential patterning of an epoxy-based negative photoresist (PR, SU-8), graphene, and MoS_2_ on an SiO_2_/Si wafer. Patterned encapsulation using SU-8 yields thick and thin regions designed specifically to aid in guiding the assembly process. Finally, a layer of photoresist (PR) serves to pattern the exposure of selected regions to ultraviolet (UV) ozone. After removing the SiO_2_ by immersion in an etchant, transfer printing with a flat slab of polydimethylsiloxane (PDMS) delivers the planar device assembly onto a film of polyvinyl alcohol (PVA) such that the topside can be exposed to UV ozone. Subsequent lamination onto a pre-stretched elastomeric substrate, pre-exposed to UV ozone, leads to strong bonding at the regions of SU-8 previously treated with UV ozone. Removing the PR layer leaves a slight physical separation between the device platform and the elastomeric substrate at corresponding regions. Releasing the substrate (Fig. 1a, right) initiates spontaneous assembly of the full 3D structure. The final architecture depends on the relevant design parameters, such as the degree of pre-stretch, the 2D geometry, and the thicknesses of the active and passive layers.

As the 3D shape evolves, the strains induced by bending and twisting must remain below the maximum endurance limit for each layer in the construct, to avoid cracking or other forms of mechanical degradation of the materials. Optimization in this context, with a goal toward realizing the desired geometry (octagonal prism, octagonal prismoid, and hemisphere), involves analytic modeling and full 3D FEA techniques that capture all details of the mechanics. The analysis starts with a precursor device design on the 2D planar surface, which subsequently morphs into a 3D structure via the process of controlled compressive buckling. Precursor designs for the octagonal prism, octagonal prismoid, and hemisphere at the 2D planar surface appear on the left side of panels b, c, and d, respectively, in Fig. 1. Various regions of the precursors adhere strongly (red) or weakly (blue) to the substrate, and some locations are relatively thin (indicated by arrow) to enable localized folding deformations. The strong and weak adhesion occurs at the strongly and loosely attached regions, respectively, of the structure after the dissolution of the PR. The pre-strain values required to form closed 3D shapes depend on the various geometric parameters of the 2D precursors (Supplementary Fig. 1). The values necessary for the cases (Fig. 1) examined here are 111, 38, and 46% for the octagonal prism, octagonal prismoid, and hemisphere, respectively. The 3D shapes evolved under these pre-strain values are in the mid-left parts of Fig. 1b–d. In particular, the design in Fig. 1d enables the formation of an approximate hemisphere, as an example of a non-developable surface that cannot be realized by simple bending/twisting deformations of an unpatterned, 2D membrane. Here, the thin regions play crucial roles, as they allow highly flexible rotation of each constituent ribbon at the outer ends adjacent to the bonding sites. Figure 1e and Supplementary Fig. 2 show that the cross-section of each intermediate state of assembly approaches an arc of evolving central angle, in which this angle increases gradually with increasing degree of release of the pre-strain, reaching 180^o^ at the final state. The heights of three mesostructures increase with the release of the pre-strain, as captured by simple analytic models (Fig. 1f, Supplementary Figs. 1 and 2). As shown in Fig. 1b–d and Supplementary Fig. 3, the strains reach peak values in the locations of the creases (indicated by arrow), where the thickness/length ratio is lower than that in the other regions. By comparison, the strains in the other parts are extremely low. The distributions of strain in the graphene and MoS_2_, separately shown in the mid-right parts of Fig. 1b–d, exhibit similar trends. The pre-strain values optimized by FEA experimentally yield 3D structures nearly identical to those predicted. The corresponding scanning electron microscopic (SEM) images are in Fig. 1b–d (right). The strain levels created in individual graphene, MoS_2_, and SU-8 layers of each 3D structure increase during the release of the pre-strain (Fig. 1g). The maximum strain in the graphene and SU-8 layers are 1.2% and ~2.3% for the octagonal prism, which are higher than those in the other structures, consistent with the comparatively high level of pre-strain required for this case. Note that the maximum strains in the graphene and MoS_2_ layers estimated by FEA are far below their corresponding elastic limits (~6 and 2%, respectively)^33–37^. The maximum strain in the MoS_2_ is ≤0.15%, indicating that MoS_2_ occupies a relatively flat region of the structure and that the strain is bending dominated. It is noteworthy that the maximum strain of MoS_2_ is much higher in the hemisphere than the other two configurations, due to the non-negligible bending deformations. The resistance of the graphene under repeated buckling and release appears in Fig. 1h. Throughout these cycles, the maximum change in resistance (~15%) occurs in the octagonal prism; changes for the octagonal prismoid and hemispherical 3D structures are relatively small. The reversible behavior observed in all three cases suggests purely elastic mechanical responses.

### Integration of 3D structure in photodetecting device and its photoresponse

**Fig. 2:**
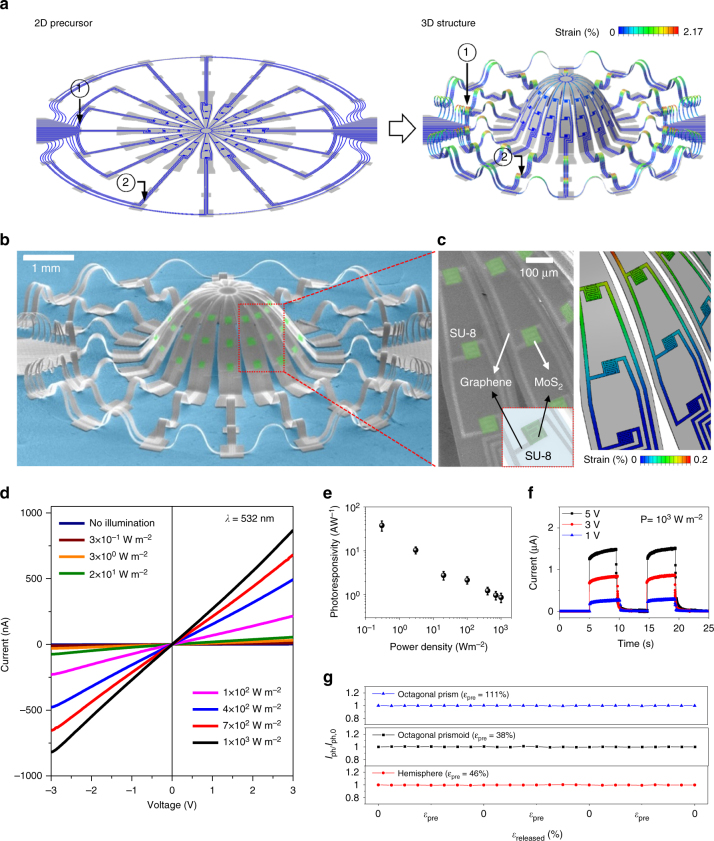
Interconnect design and photoinduced response of MoS_2_ photodetectors on a 3D hemispherical structure. **a** FEA of the 3D hemisphere structures before and after compressive buckling, showing the distributions of maximum principal strains in the MoS_2_ photodetectors and 3D interconnects. **b** Colorized SEM image of MoS_2_ photodetectors consisting of MoS_2_ (green), graphene (light gray), and SU-8 (gray) on the hemisphere with 3D interconnects. **c** Magnified view of the SEM image in **b** and corresponding FEA results for the strain distribution. Inset: schematic illustration of the unit device enclosed by the red box in the main image. **d** *I*–*V* characteristics of the 3D photodetector at different bias voltages (laser-beam wavelength = 532 nm, power density = 1000 W m^−2^). **e** Photoresponsivity of the MoS_2_ photodetector at a bias voltage of 3 V for different laser power densities. (Standard deviation of 48 sampling distribution recorded from 48 devices.) **f** Time-resolved photoresponses of the devices under laser illumination at different power densities. **g** Variation in photocurrent during repeated buckling processes for the octagonal prism (top), octagonal prismoid (middle), and hemispherical structure (bottom)

System-level operation requires electrical interconnects to external data acquisition equipment, with designs capable of accommodating the 3D transformation and associated buckling processes. Figure 2a shows a 2D precursor that satisfies these requirements, along with the corresponding 3D structure that results after releasing the pre-strain (46%) (Supplementary Movie 1). These traces (as well as interconnects in the active regions of the device) exploit bi-layer graphene sandwiched between two SU-8 layers. As with main part of the system, selective bonding regions result from patterned UV ozone exposure. These traces evolve into their own 3D shapes, in parallel with the central hemisphere. The maximum strain (2.17%) appears in the regions of smallest bending radius, designated as 1 (circled, Fig. 2a). Supplementary Fig. 4 presents the strain distribution for two designated points 1 and 2 located on the interconnects. The maximum strains, as estimated in simulations, are again far below the intrinsic elastic limit of graphene (~6%)^33–35^. The areal proportion of the regions that undergo significant strain (>2%) is less than 0.2% (Supplementary Fig. 5). Figure 2b presents an SEM image of the resulting 3D system. The hemispherical structure supports three MoS_2_ photodetectors on each arm, for a total of 48 devices in the entire array, all interconnected with a network of bi-layer graphene traces (Fig. 2c shows a magnified view of SEM (left) and simulated (right) parts of the structure). The resistance of the graphene in the regions of lowest bending radius (where the strain is maximized at 2.17%) is ~20.7 kΩ vs. ~18.3 kΩ in the flat geometry (Supplementary Fig. 6). Such slight variations in resistance have a negligible effect on the overall operation, as they are much lower than the resistance of the photoactive material MoS_2_ (~1 MΩ).

Visible light (532 nm) passed over a representative photodetector induces a photoresponse that can be captured as current–voltage (*I*–*V*) characteristics for different illumination intensities (Fig. 2d). The photocurrent (*I*_ph_) increases with intensity and with bias voltage, in a manner that is symmetric around 0 V. The linear behavior and symmetric *I*–*V* characteristics are consistent with approximately ohmic contacts between the graphene and MoS_2_ (Supplementary Fig. 7). The maximum ratio of *I*_ph_ in illuminated and dark states reaches ~427 at an intensity of 10^3^ W m^−2^ (Supplementary Fig. 8). The photoresponsivity, calculated as *R*_ph_=*I*_ph_/*P*_in_, where *I*_ph_ is the photocurrent and *P*_in_ is the incident laser power, is 38 A W^−1^ at an intensity of 0.3 W m^−2^, and decreases with increasing intensity (Fig. 2e). These observations are qualitatively and quantitatively comparable to the *R*_ph_ values previously reported in MoS_2_-based photodetector devices^27–32^. Time-resolved measurements at different bias voltages (Fig. 2f) indicate that the device responds to the “on” and “off” switching of the laser beam, and its corresponding temporal *I*_ph_ is quite uniform (1.49, 0.84, and 0.28 µA corresponding to bias voltages of 1, 3 and 5 V, respectively, at 1000 W m^−2^ power density) through multiple cycles at each bias voltage^27–32^. The estimated rise (*τ*_rise_) and decay (*τ*_decay_) times of the *I*_ph_ are 290 ± 130 ms and 420 ± 210 ms, respectively,^27,28,31^. The time-resolved photoresponses are lower than those of similar devices fabricated on SiO_2_ substrates (Supplementary Fig. 9), possibly due to the larger number of surface traps at the MoS_2_/SU-8 interface than at the MoS_2_/SiO_2_ interface^30,38^. The detection mechanism is based on photoresistive behavior, with a response time that is slower than that of devices such as photodiodes or phototransistors^30,32^. The photodetectors responded 20 times faster when encapsulated with the sandwich structure: Al_2_O_3_/MoS_2_/Al_2_O_3_. The high-k dielectric Al_2_O_3_ layer significantly reduces interface trap charges and results in a clean, conformal and low roughness interface that also efficiently suppresses Coulombic impurities (Supplementary Fig. 10)^39,40^. For all cases, the 3D structures (the octagonal prism, octagonal prismoid, and hemisphere) can be reversibly stretched and deformed from 2D to 3D states; the corresponding stability behaviors, as in Fig. 2g, are consistent with robust operation under the high strain levels and cycling. Further, the 3D photodetectors are environmentally stable (Supplementary Fig. 11) and can be formed in high density layouts (up to 10,000 on a single hemispherical surface) across arrays of separate 3D devices that can all be assembled in a single step. The mechanical flexibility of the supporting substrate and the intrinsic deformability of the 3D structures allow such systems to be bent, stretched and twisted in a reversible, non-destructive fashion (Supplementary Figs. 12−14 and Movie 2). These features in stability, manufacturability, and deformability suggest potential for use in practical applications with unique modes of operation.

In combination, the assembled layers in these photodetecting devices (the bi-layered graphene, monolayer MoS_2_, and the 7.5-μm SU-8 layer) exhibit high optical transmittance (~87%) at 550 nm (Supplementary Fig. 15). An array in a 3D format can therefore detect the position and intensity of illuminating light simultaneously, in a manner that cannot be replicated easily with traditional photodetector arrays or those in 2D layouts. Current 2D solutions involve external light-blocking screens (e.g., a wall and a cylinder with a pinhole or a slit), a pair of actuators, and a driving motor connected with microprocessor^41,42^. Although functional, this multicomponent apparatus cannot be easily scaled to small dimensions. By contrast, the transparency and 3D shape of the systems introduced here allows light to pass through the entire device, to provide measurements both at the point of illumination but also at the point where the light passes out of the structure. The results allow collection of beam directionality and divergence.

### Mechanism, principle and determination of position and direction of incident light

**Fig. 3:**
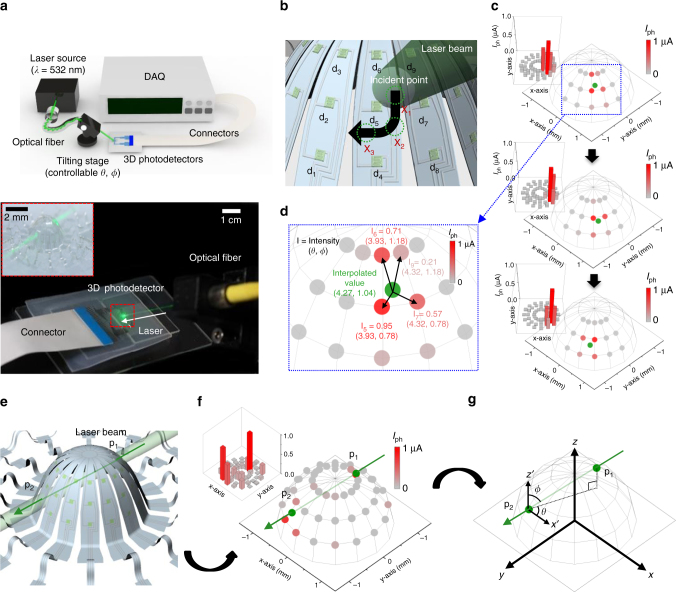
Operating principles of a 3D photodetection and imaging system. **a** Schematic illustration and optical images of the system. Inset: magnified view. **b** Distribution of the devices and movement of the laser beam. **c** Photocurrent distribution on the hemisphere surface during movement of the laser beam. **d** Magnified view of the photocurrent distribution at the first position of the laser beam. The illuminating locations of the incident laser beam are estimated by interpolation. **e** Penetration of the laser beam at two points of the 3D hemispherical surface. **f** Photocurrent distribution on the hemisphere surface in the scenario of **e**. **g** Principle of estimating the incident direction of the laser from the photocurrent map

The system in Fig. 3a allows for the demonstration of these capabilities. Here, a laser source (532 nm) and goniometer allow motion of the laser beam in 3D coordinates, to provide incidence at any desired position. An automated measurement unit records the photoresponses of the 48 devices with ~64 Hz device interval (corresponding frame interval ~0.75 s), as in the bottom of Fig. 3a. The devices nearest to the location of illumination (entry or exit point) exhibit the highest photoresponses; those of the other devices decrease with increasing distance from this location owing to scattered light (Supplementary Fig. 16). Only the nine devices nearest to the illumination point respond significantly to the light signal; therefore, the position of the incident light is computed from the known coordinates of these nine devices (with respect to the center of the hemisphere). The calculation is according to

1 $${{P}}_{\mathrm{I}}({\mathrm{\theta }}_{\mathrm{I}},{\mathrm{\varphi }}_{\mathrm{I}}) = (\mathop {\sum }\limits_1^9 (\theta _n \ast \frac{{I_{{\rm ph},n}}}{{\mathop {\sum }\nolimits^ I_n}}),\,\mathop {\sum }\limits_1^9 (\varphi _n \ast \frac{{I_{{\rm ph},n}}}{{\mathop {\sum }\nolimits^ I_n}})),$$

where *P*_I_ is the spherical coordinate of the incident point, *I*_ph_ is the photocurrent for the *n*th device, and *θ* and *φ* are the azimuthal and polar angles, respectively. As the laser beam moves, its trajectory can be recorded in this manner, as indicated by the arrow in Fig. 3b. From the initial point X_1_ (Fig. 3b), a continuous photoresponse spectrum immediately begins up to the final point X_3_. The responses of the nine nearest photodetecting devices in the recorded data define the location of the beam. Figure 3c shows the estimated positions of the beam (green dot) and the photoresponses of the nearest devices at three locations X_1_, X_2_, and X_3_ (Fig. 3b). These measured movements exactly match the laser movements, as indicated by the arrow (Fig. 3b). The device closest to the incident point delivers the maximum photoresponse (bright red point). Figure 3d shows the interpolated position of the incident laser beam corresponding to location X_1_ in spherical coordinates originating at the hemisphere center.

### Real-time imaging of incident light

**Fig. 4:**
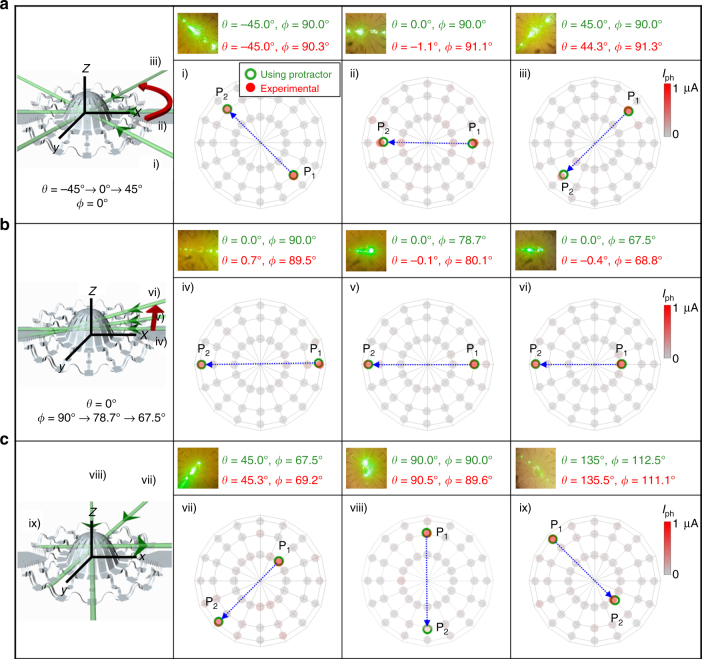
Sensing characteristics of a 3D photodetection and imaging system. **a** Maps of photocurrent measured across the 3D surfaces. The incident angle *θ* ranges from −45° to 45° with *φ* fixed at 90°. The measured *θ* and *φ* from the device arrays are shown for comparison. **b** Similar results with *φ* ranging from 90° to 67.5° and *θ* = 0°. **c** Photocurrent maps of the photodetector array on the 3D surfaces randomly penetrated at two points by the incident laser. The incident angles are (from left to right) *θ* = 45°, *φ* = 67.5°, *θ* = 90°, *φ* = 90°, and *θ* = 135°, *φ* = 112.5°

As mentioned previously, the passage of the laser beam through the transparent 3D system provides additional locations for determining the direction of the beam: an exit (P_2_) in addition to an entry (P_1_) (Fig. 3e). Figure 3f shows the mapping result for a representative case. As indicated in the bar graphs, the photoresponses of devices nearest to P_1_ are slightly stronger than those at P_2_, due simply to partial reflections and absorption (the device surface transmits 87% of the incident light). The direction of the incident beam (P_1_ → P_2_) follows from the spherical locations of the P_1_ and P_2_ vectors with respect to the hemisphere center. The P_1_ → P_2_ direction can be defined in terms of the azimuthal angle *θ* in the *x*–*y* plane and the polar angle *φ* between the *x*–*y* plane and *z* direction (Fig. 3g). This simple example extends naturally to more complex cases involving movement of the laser beam in *θ*, *φ*, and both planes. For present purposes, we explore three scenarios: (1) increasing *θ* from −45.0° to 45.0° while fixing *φ* at 90.0°, (2) decreasing *φ* from 90.0° to 67.5° while fixing *θ* at 0.0°, and (3) increasing *θ* from 45.0° to 135° and *φ* from 67.5° to 112.5°. In each, three combinations of *θ* and *φ* are tested, as shown in Fig. 4, with the laser beam incident on top of the 3D system. The direction (P_1_ → P_2_) can be expressed in spherical coordinates *θ* and *φ*. In all combinations in the three scenarios (nine experiments in total), the *θ* and *φ* coordinates calculated from the measured photoresponses match the coordinates directly measured with a protractor scale (Supplementary Fig. 17), as summarized in panels (a)–(c) of Fig. 4 along with the spatial mapping of the photoresponse. Corresponding bar graphs are in Supplementary Fig. 18. Note that although the system can map 360° rotations of *θ* in the *x*−*y* plane, the *φ* movement is limited by the array geometry, which imposes lower and upper circumferences on the hemispherical surface; see Supplementary Fig. 19. Simple modifications allow up to 45° movement of *φ* (Supplementary Fig. 20). All measurements and analysis can be performed in real time, as shown in the video clip of the supporting materials (Supplementary Movie 3). Further, the degree of accuracy in determining the position and direction improves with decreases in the distances between photoresistors and in the laser spot sizes (Supplementary Figs. 21 and 22). Moreover, the representative 3D photodetector showed good imaging capability in kHz range (Supplementary Fig. 23).

## Discussion

Exploiting MoS_2_ and graphene in complex 3D system architectures allows their unique electronic, mechanical, and optical properties to be leveraged in a photodetection and imaging device that can sense both the direction and intensity of illumination, in real time. Similar capabilities cannot be achieved easily, with solid state, integrated operation, by using conventional designs or materials. Key enabling properties of 2D materials in this context include high threshold strains, to avoid fracture even in extreme cases of 2D to 3D transformation, ultrathin geometries, to minimize bending-induced strains that follow from this transformation and optical transparency, to allow simultaneous illumination on the front and backsides of the system. The example presented here might foreshadow other opportunities to engineer unique system-level function by deploying 2D materials in 3D designs.

## Methods

### Growth of 2D materials

Monolayer graphene and MoS_2_ were grown on a Cu foil and a SiO_2_/Si wafer, respectively, by the low pressure chemical vapor deposition and the metalorganic chemical vapor deposition (MOCVD). The Cu foils (16 × 8 cm^2^) were inserted in a circular quartz tube and thermally annealed up to 1000 °C in the presence of H_2_ gas (8 sccm) at 80 mTorr for 2 h. The chamber was then injected with CH_4_ precursor gas (20 sccm) at 1.6 Torr for 1 h. Subsequently, the quartz tube (heated zone) was allowed to naturally cool at an initially rapid rate, before being gradually raised to room temperature in the presence of H_2_ gas (8 sccm) at 80 mTorr. Chemical precursors to Mo and S for growth of MoS_2_ were molybdenum hexacarbonyl (MHC) and dimethyl sulfide (DMS), respectively. The gas phase MHC (0.5 sccm) and DMS (1 sccm) were inserted into the MOCVD quartz tube along with H_2_ (10 sccm) and Ar (300 sccm), and the tube was heated to 550 °C at 7.5 Torr for 20 h. Raman and photoluminescence (PL) results from MoS_2_ and graphene are shown in Supplementary Figs. 24 and 25, respectively. The results confirm that MoS_2_ is monolayer and graphene is double-layer.

### Transfer process of 2D materials

Polymethyl methacrylate (PMMA) was spin-coated on top of the graphene of one side of the Cu foil (the other side was etched out by O_2_ plasma) and on the MoS_2_ monolayers to provide support during the transfer process. The Cu foil was etched by floating the PMMA-coated graphene/Cu foil on ammonium persulfate (APS) solution (20 g ℓ^−1^) for 5 h. After etching the Cu foil, the PMMA/graphene film was floated multiple times on deionized (DI) water to completely wash away the APS residue. Finally, the graphene was transferred to the desired wafer and the PMMA was removed by acetone. The SiO_2_ on the MoS_2_ was etched by floating the PMMA-coated MoS_2_ SiO_2_/Si wafer on diluted (1%) hydrogen fluoride (HF) solution. Afterward, the HF residues were washed away by floating the PMAA/MoS_2_ film on DI water in a manner similar to that for the PMMA/graphene film.

### Fabrication of 3D photodetectors

First, SU-8 (2 µm) was spin-coated on an SiO_2_/Si wafer and patterned according to the desired 3D structure (octagonal prism, octagonal prismoid, or hemisphere). After transferring the graphene, the electrodes were defined in an interdigitated geometry by photolithography and reactive ion etching (RIE) with O_2_ plasma (40 sccm, 100 W, 5 s). MoS_2_ was transferred onto the interdigitated pattern of graphene electrodes and the channel regions were defined by photolithography and RIE with CHF_3_/O_2_ plasma (35/10 sccm) at 100 W for 5 s. Another SU-8 (5 µm) layer was spin-coated and patterned in a manner similar to that for the first SU-8 layer, but with openings in regions designated for creases. PR was spin-coated and patterned to expose and cover the bonding and non-bonding regions, respectively. Assisted by HF treatment, this fabricated structure was transferred with a slab of PDMS to a tape of PVA. Next, an elastomer substrate (Dragon Skin, Smooth-On) was bi-axially pre-stretched to the optimized strain determined by FEA simulations. The structure on the PVA tape and the pre-stretched substrate were exposed to UV ozone, laminated together and then baked in an oven at 70 °C for 5 min. The PVA was then dissolved in DI water. Finally, the PR was dissolved in acetone, which loosened and/or slightly delaminated the non-bonding regions of the devices from the pre-stretched substrate, facilitating the 3D assembly process upon release of the pre-stretching strain. The sizes of MoS_2_ photoresistor and the 3D pop-up hemisphere structure are 80 × 80 µm and 3.5 × 1.4 mm (diameter × height), respectively. Detailed descriptions of the 3D hemispherical structure and diagrams of the operating principles are in Supplementary Figs. 26 and 27, respectively.

### Finite element analyses

Three-dimensional FEA allowed prediction of the mechanical deformations and strain distributions of photodetector structures and the entire circuit system enabled by controlled buckling. Eight-node 3D solid elements and four-node shell elements with a multiple stack design (SU-8/MoS_2_/Graphene/SU-8 or SU-8/Graphene / SU-8) were used to model the silicone substrate and 2D precursors, respectively. Refined meshes of those elements ensured the computational accuracy. The critical buckling strains and corresponding buckling modes determined from linear buckling analyses were implemented as initial imperfections in the postbuckling calculations to obtain the deformed configurations and strain distributions during the pre-strain release. The simulations of postbuckling process were performed using conventional static analysis in the commercial software ABAQUS. The elastic modulus (*E*) and Poisson’s ratio (*ν*) are *E*_substrate_ = 166 kPa and *ν*_substrate_ = 0.49 for substrate; *E*_graphene_ = 500 GPa and *ν*_graphene_ = 0.15 for graphene; ${\it{{E}}}_{{\mathrm{MoS}}_2}$ = 270 GPa and $\nu _{{\mathrm{MoS}}_2}$ = 0.25 for MoS_2_; and $E_{{\mathrm{SU}} - 8}$ = 4.02 GPa and $\nu _{{\mathrm{SU}} - 8}$ = 0.22 for SU-8.

### Measurement of photoresponse

A laser source of wavelength 532 nm and the diameter of ~315 µm was connected to a tilting stage through an optical fiber. The tilting stage enabled easy adjustment of the incident direction of the laser beam. Electrical measurements of the photodetecting device were performed using a semiconductor characterization system (Keithley 4200). The photoresponse was recorded in real time by using a data acquisition system (DAQ) (Keithley 3706A) and a source meter (Keithley 2612). The raw photoresponse data from the DAQ were input into a program (encoded in MATLAB) to allow visualization of the photocurrent mapping. The program represents the intensity of the photocurrent mapping by a color scale and calculates the direction based on Eq. (1).

### Data availability

Data supporting the findings of this study are available within the article and its Supplementary Information file, and from the corresponding authors upon reasonable request.

## Electronic supplementary material

Supplementary Information

Description of Additional Supplementary Files

Supplementary Movie 1

Supplementary Movie 2

Supplementary Movie 3
