# Supplementary material for: Two-dimensional materials in functional three-dimensional architectures with applications in photodetection and imaging
Source: Nat Commun. 2018 Apr 12;9:1417. doi: 10.1038/s41467-018-03870-0 (PMC5897379; doi:10.1038/s41467-018-03870-0)
Supplement: Supplementary file 1 — Supplementary Information [file 41467_2018_3870_MOESM1_ESM.pdf]

Supplementary Information

Two-Dimensional Materials in Functional Three-Dimensional Architectures with Applications in Photodetection and Imaging

Wonho Lee[1,†], Yuan Liu[2,†], Yongjun Lee[1,†], Bhupendra K. Sharma[1], Sachin M. Shinde[1], Seong Dae Kim[1], Kewang Nan[3], Zheng Yan[3], Mengdi Han[3], Yonggang Huang[4], Yihui Zhang[2], Jong-Hyun Ahn[1]* & John A. Rogers[3]*

[1]School of Electrical and Electronic Engineering, Yonsei University, 50 Yonsei-ro, Seoul 03722, Republic of Korea

[2]Department of Engineering Mechanics, Center for Mechanics and Materials and Center for Flexible Electronics Technology, AML, Tsinghua University, Beijing, 100084, China.

[3]Departments of Materials Science and Engineering, Biomedical Engineering, Chemistry, Mechanical Engineering, Electrical Engineering and Computer Science, Center for Bio-Integrated Electronics, Simpson Querrey Institute for Nano/Biotechnology, Northwestern University, Evanston, Illinois 60208, USA.

[4]Departments of Civil and Environmental Engineering, Mechanical Engineering, and Materials Science and Engineering, Northwestern University, Evanston, IL 60208, USA.

[†] These authors contributed equally to this work.

*Corresponding authors: ahnj@yonsei.ac.kr and jrogers@northwestern.edu

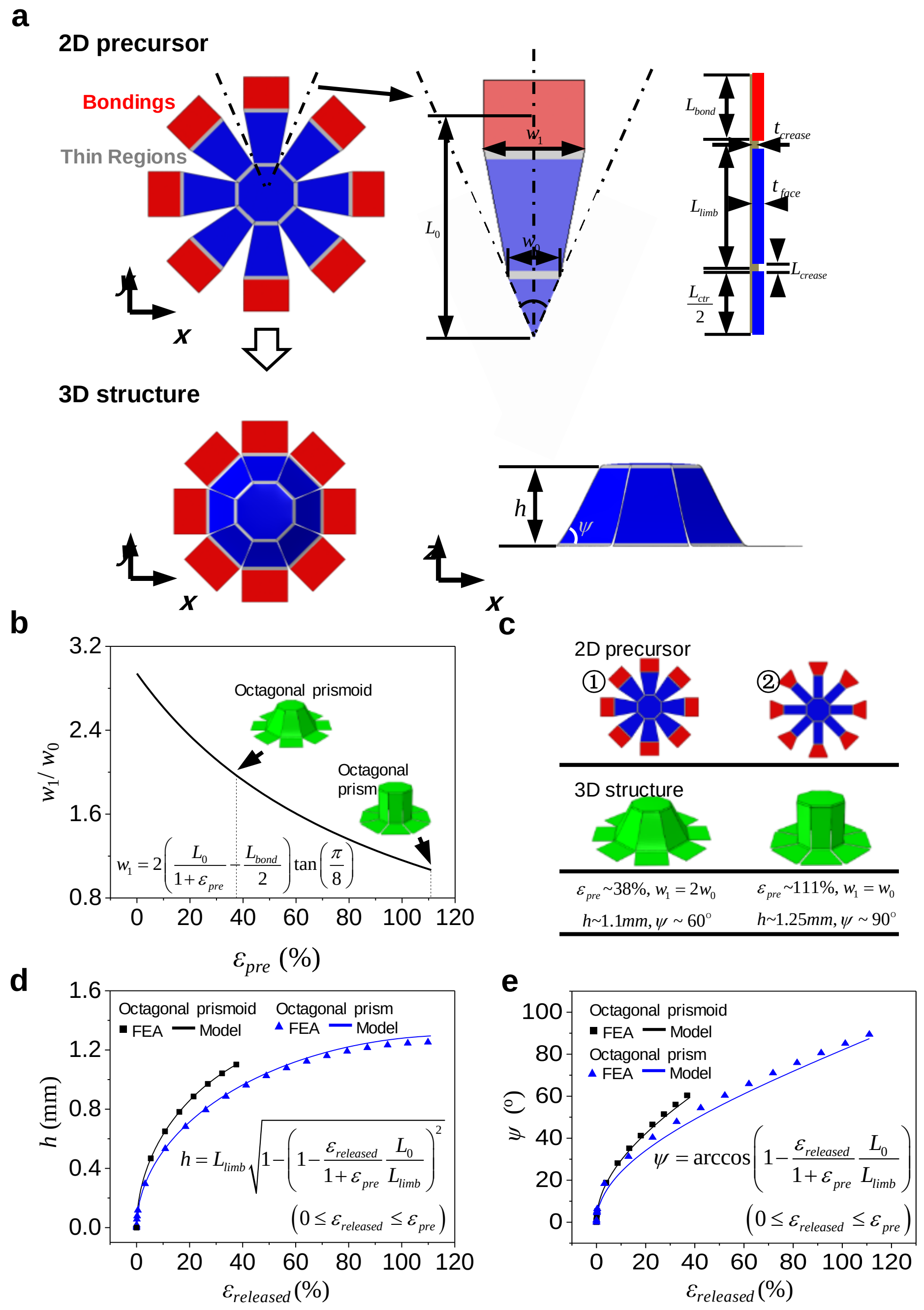

Supplementary Figure 1| Modeling of 3D structure. a, Illustration of the various geometric parameters associated with the 2D precursor and the final 3D shape. **b**, The design parameter (width ratio w_1/w_0) versus the pre-strain value required to form a closed prismatic shape after 3D assembly. **c**, Key design parameters and shape parameters of two representative prismatic structures studied in Figure 1. **d**, Analytic predictions and FEA results of the height of the prismatic structures as a function of the released pre-strain. **e**, Analytic predictions and FEA results of the tilted angle of the prismatic structures as a function of the released pre-strain.

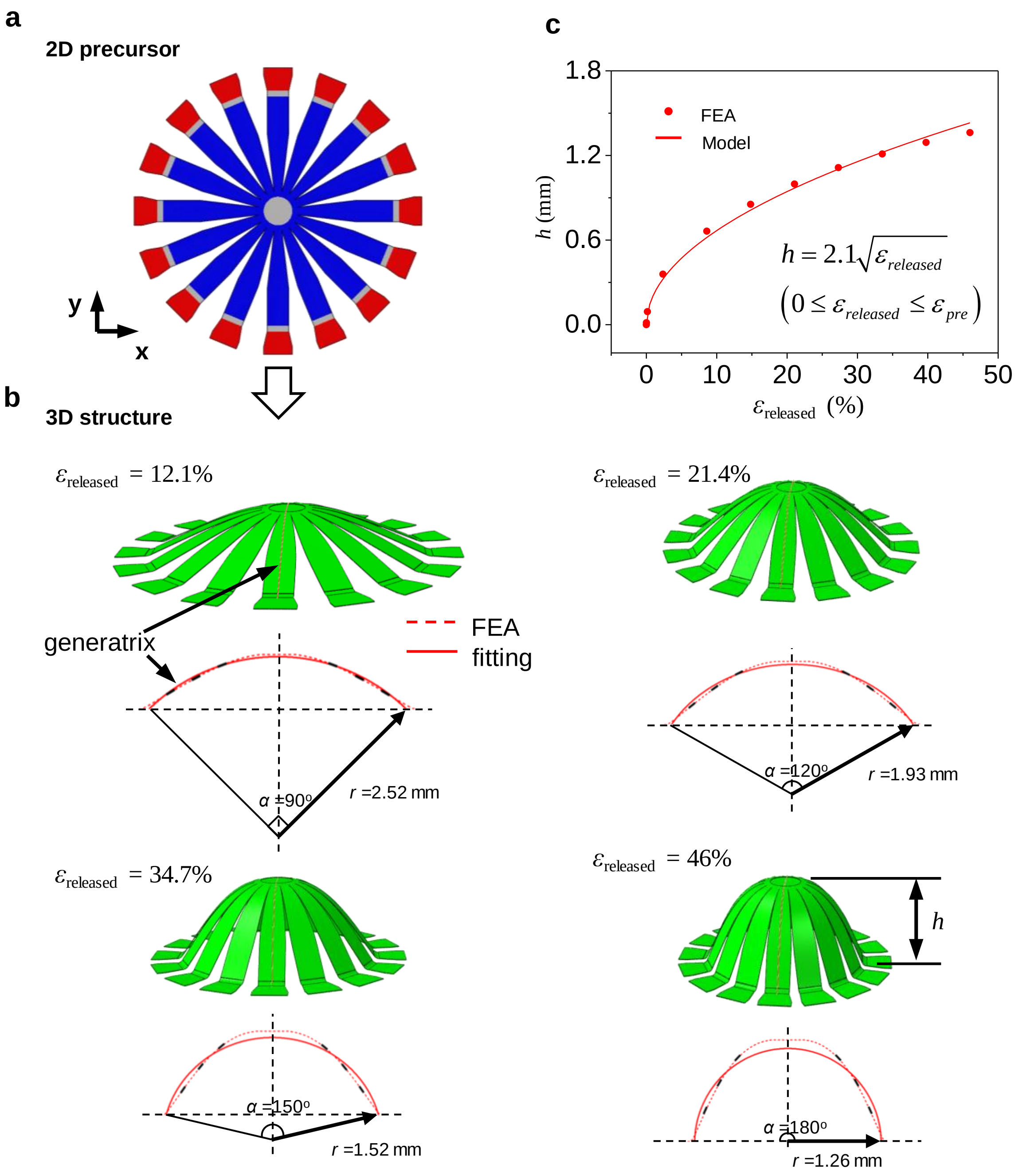

Supplementary Figure 2 | Intermediate states in the evolution of 3D shape against releasing the pre-strain. a, Schematic illustration of the 2D precursor for the hemisphere photodetector. **b**, 3D configurations and their cross sections at the intermediate and final states of mechanical assembly. In the cross-sectional views, the dashed and solid lines correspond to the FEA results and the fitting curves using ideal arcs, respectively. **c**, Analytic predictions and FEA results of the mesostructure height as a function of the released pre-strain.

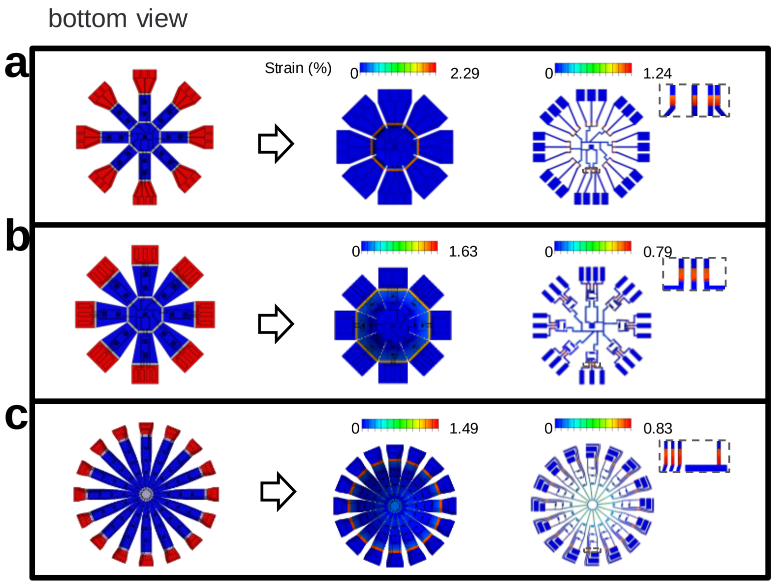

Supplementary Figure 3 | Strain distribution on surface of evolved 3D structures. Distributions of the maximum principal strain from the bottom view for the three photodetector structures in Figure 1. The peak value of maximum principal strain appears at the bottom-side surface of the crease region.

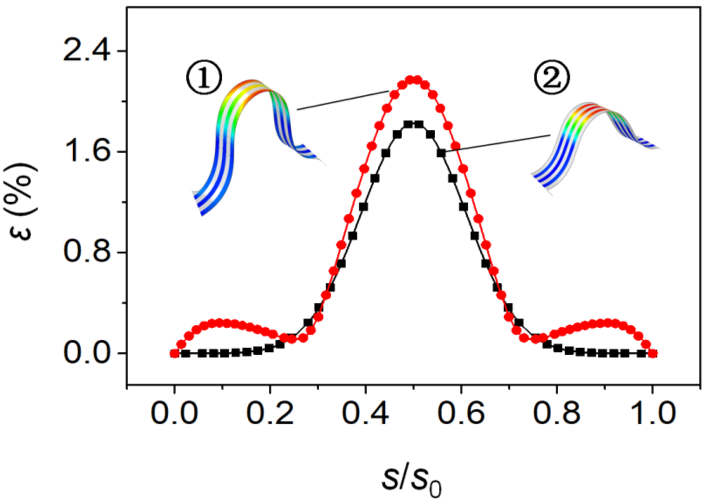

Supplementary Figure 4 | Strain profiles of interconnects. Strain profiles of two designated points ① and ② of interconnects.

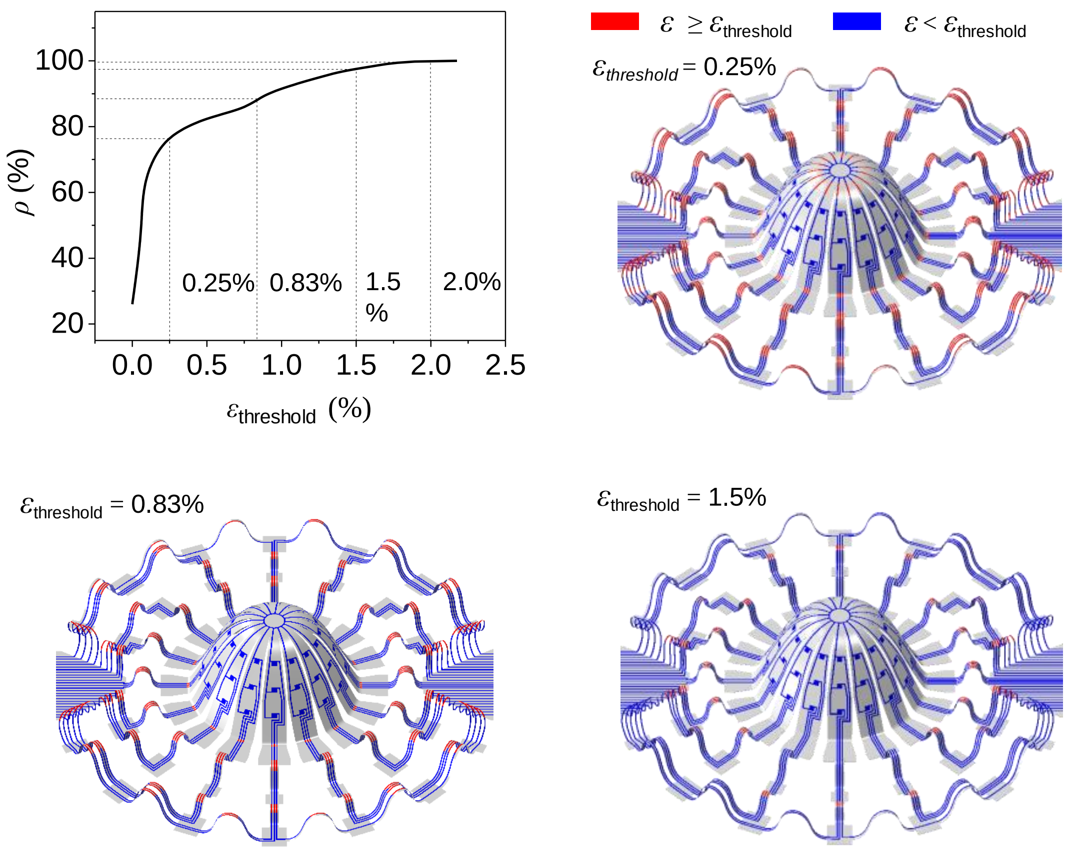

Supplementary Figure 5 | Areal proportion of strained regions (> 2%). The areal proportion (ρ) of the interconnects whose maximum cross-sectional strain is below a threshold value ($\varepsilon_{\text{threshold}}$). The three images highlight the relatively high strain region as compared to three different threshold values.

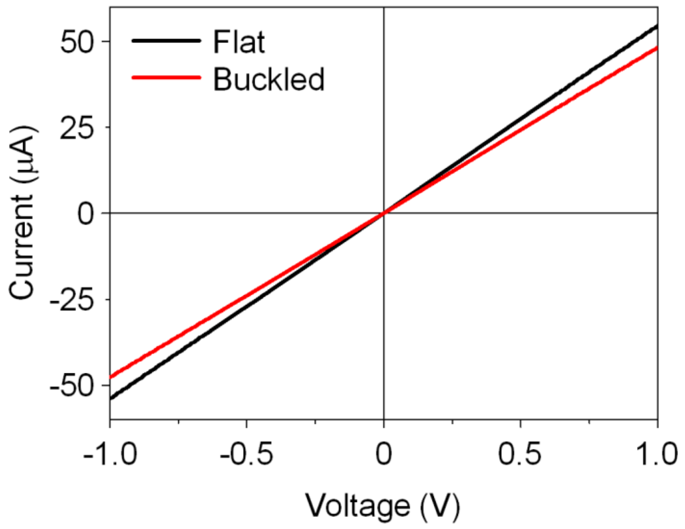

Supplementary Figure 6 | Resistance of graphene in flat and maximum strain regions. *I*-*V* characteristics of the flat and the buckled graphene/SU-8 interconnects.

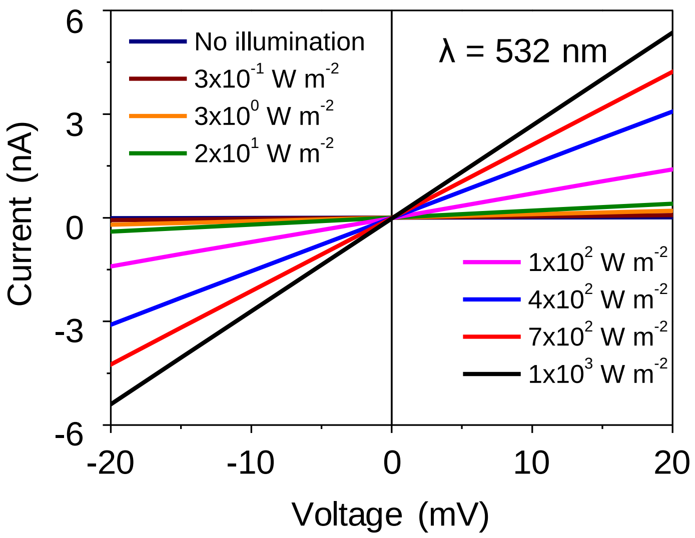

Supplementary Figure 7 | Photoresponse of 3D MoS_2 photodetector at different illuminating powers. *I*-*V* characteristics of the MoS_2 photodetectors in the low voltage range from -20 to 20 mV.

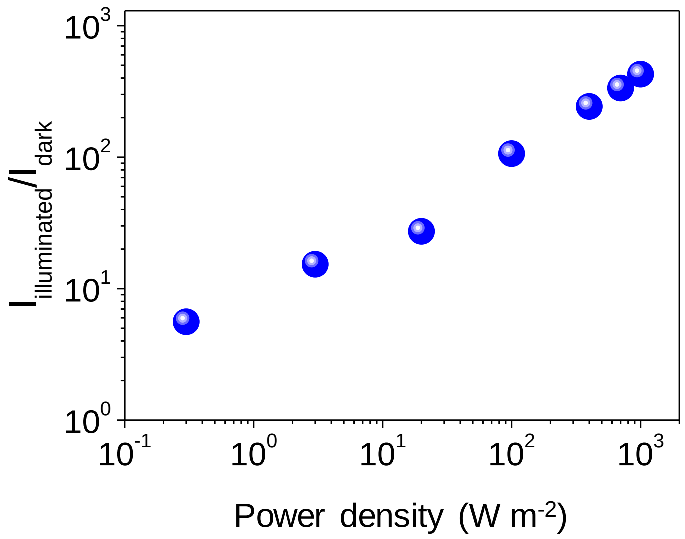

Supplementary Figure 8 | On/Off current ratio of 3D MoS_2 photodetector. Current ratio of the dark and illuminated states of the MoS_2 photodetectors measured with different values of the laser power density.

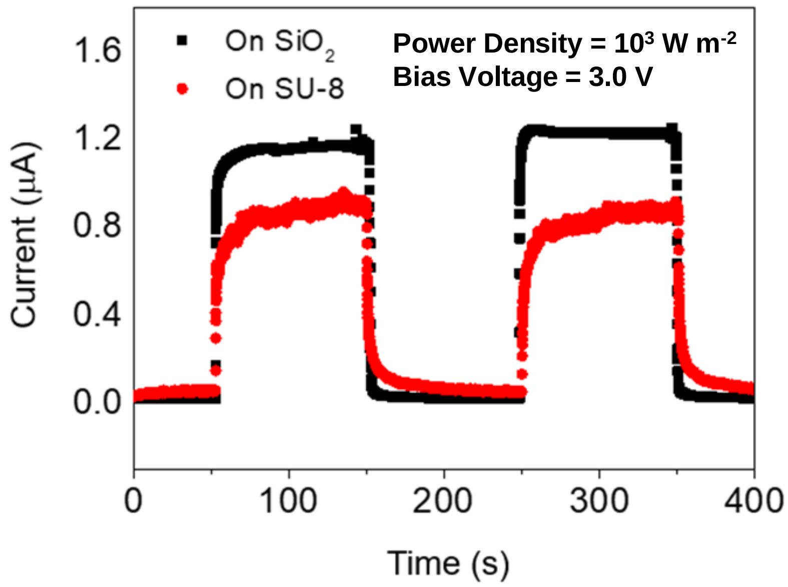

Supplementary Figure 9 | Temporal photoresponses. Comparison of time-resolved photoresponse of the MoS_2 photodetectors on SiO_2 and SU-8.

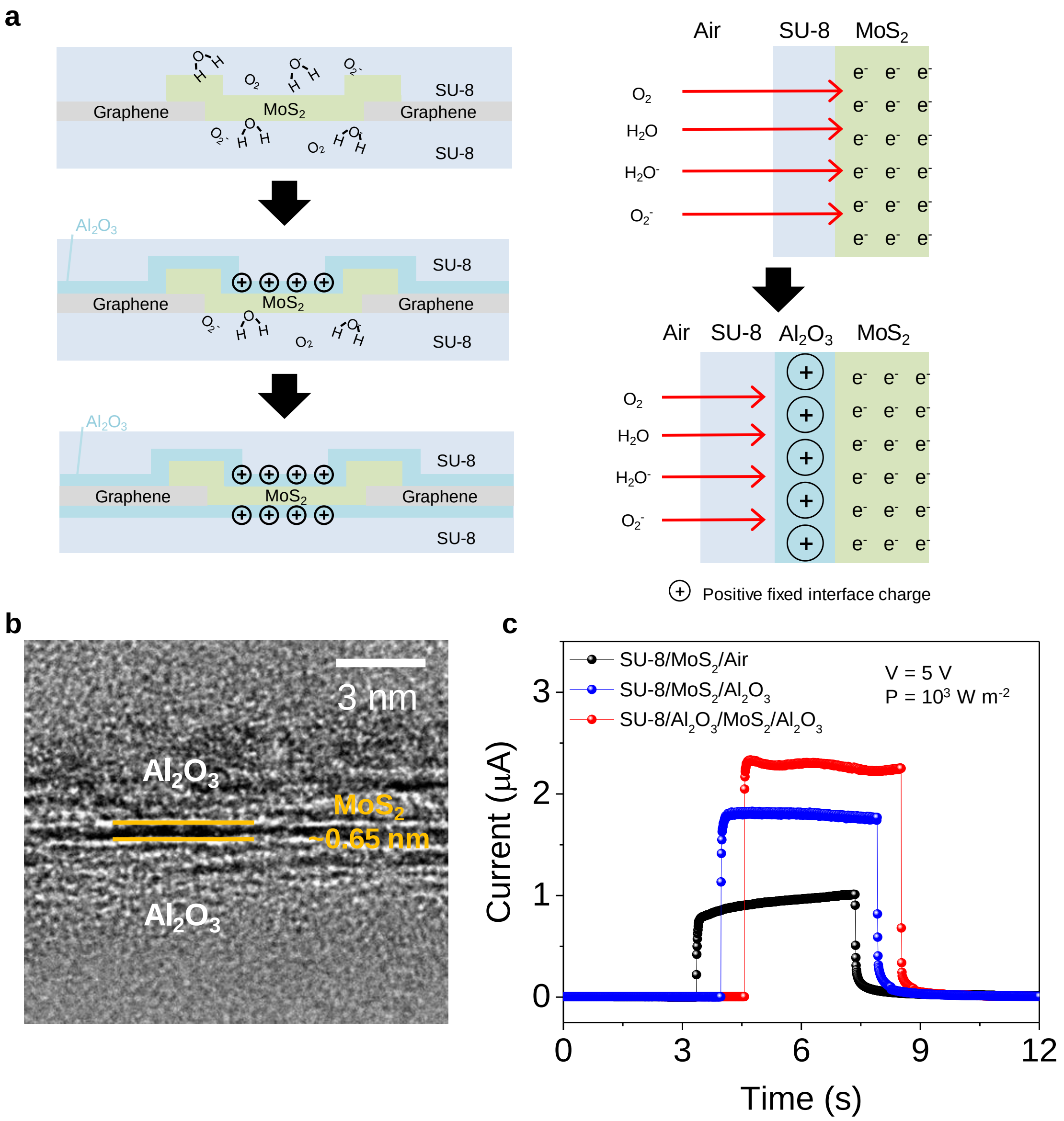

d

	τ_{rise} (ms)	τ_{decay} (ms)
SU-8/MoS_2/Air	176.7±95.4	264.9±120.7
SU-8/MoS_2/Al_2O_3	29.52±17.4	51.66±22.7
SU-8/Al_2O_3/MoS_2/Al_2O_3	7.3±4.2	14.7±7.5

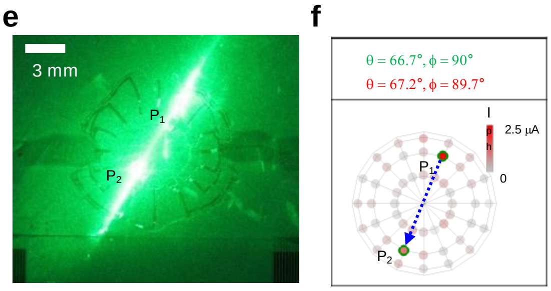

Supplementary Figure 10 | Photoresponse enhancement in Al_2O_3 sandwiched MoS_2 3D photodetector. a, Schematic illustrations of devices in cross-sectional view (left) displaying trapped water (H_2O) and oxygen (O_2) molecules without Al_2O_3 and the elimination of such traps with insertion of Al_2O_3 at the MoS_2 interfaces. The traps deplete the carriers in MoS_2 (right, top); insertion of Al_2O_3 reduces such effects. **b**, Cross-sectional transmission electron

microscope image of a $Al_2O_3/MoS_2/Al_2O_3$ sandwich structure, highlighting the physical nature of the Al_2O_3/MoS_2 interfaces. **c**, The photoresponses of a photoresistor without encapsulation, with top Al_2O_3 encapsulation, and with top and bottom (sandwich) Al_2O_3 encapsulation. **d**, The rise and decay time clearly reveal a significant increase in response speed (20 times) with the addition of high-k dielectric encapsulation, as compared to the unencapsulated case. **e**, Optical photograph of 3D photodetector with a sandwiched structure and **f**, corresponding photoimaging results. The high-k Al_2O_3 layer provides a clean, conformal contact, and imparts low surface roughness to the MoS_2 interface, thereby reducing the interface traps. Additionally, the large dielectric mismatch between Al_2O_3 and MoS_2 suppresses Coulombic impurities in MoS_2, further decreasing the carrier trapping during transport. The effects facilitate fast carrier transport upon illumination, thus increasing the response speed.

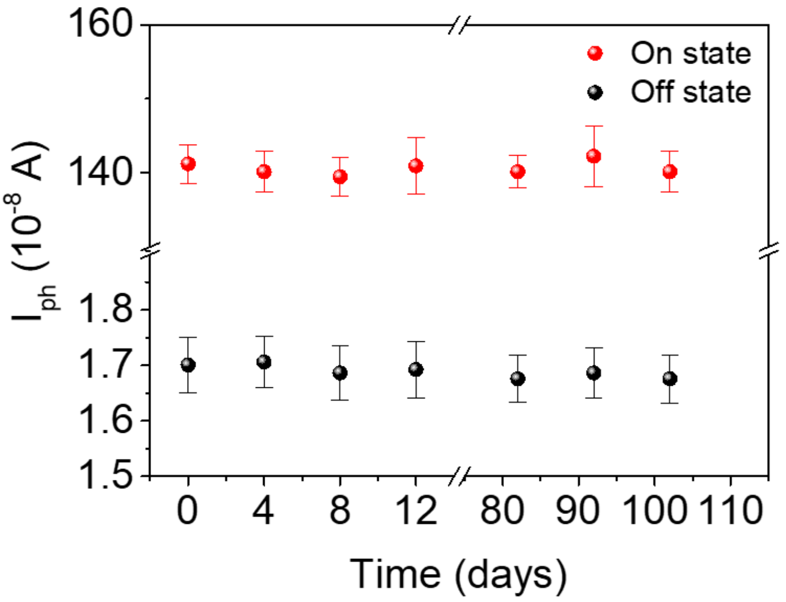

Supplementary Figure 11 | Stability test in ambient environment. The dark current and photoresponse(Standard deviation) remain unchanged over > 3 months (the duration of the test), indicating excellent environmental stability and consequent potential for use in practical applications. The stable operation follows partly from the chemically inert nature of the constituent 2D materials; graphene and MoS_2.

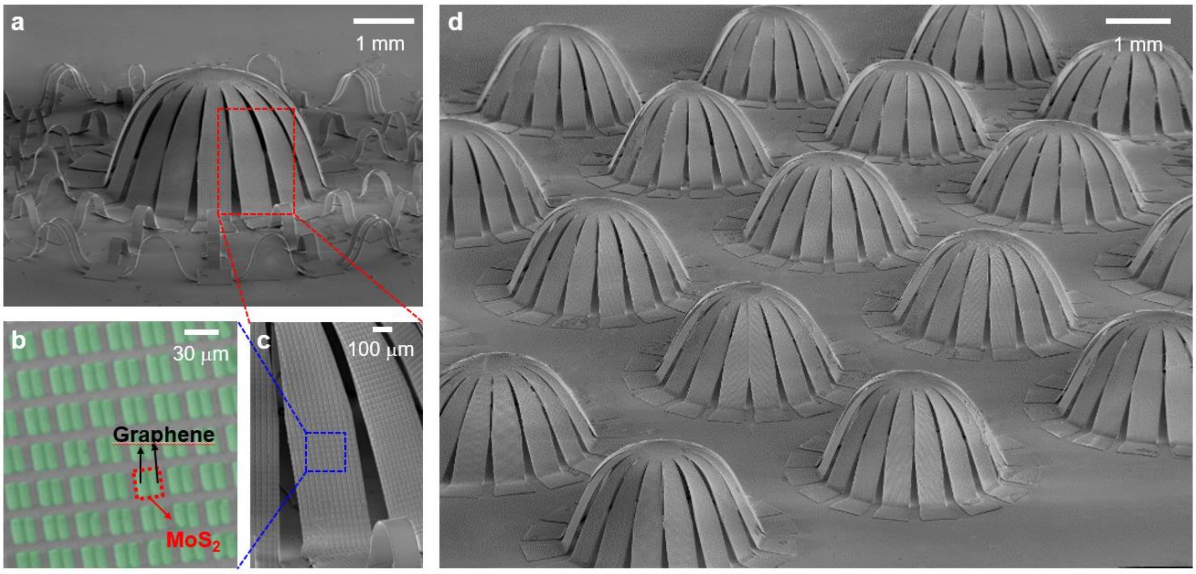

Supplementary Figure 12 | SEM image of scalable, parallel formation of an array of 3D photodetection systems. Each incorporates 10,000 individual photoresistor units (16 arms, each arm accommodating ~6,000 photoresistors) on its surface (**a**, **b** and **c**). These densely packed devices follow from a single assembly step, from a collection of 2D precursors fabricated in parallel using planar processing techniques. The scalability in resolution and the parallelism of the manufacturing approaches are consistent with use in practical applications. **d**, SEM image of a 5x5 array of such 3D structures.

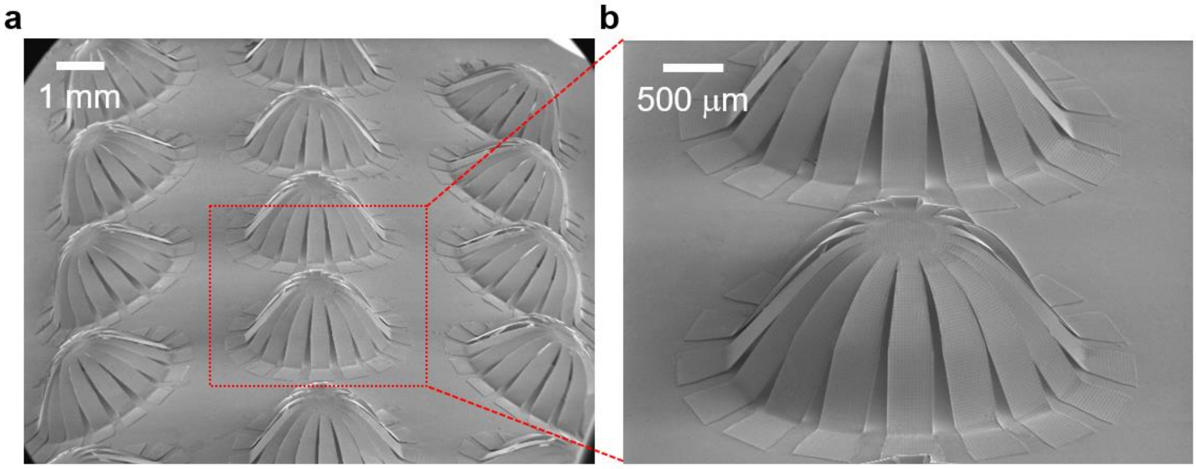

Supplementary Figure 13 | SEM mapping under bent states. a, SEM image of an array of 3D photodetector systems was recorded in a bent configuration (bending radius 1 cm) **b**, Magnified view of the center part in a bent configuration. The absence of any evidence of delamination or cracking indicates its excellent mechanical endurance during bending.

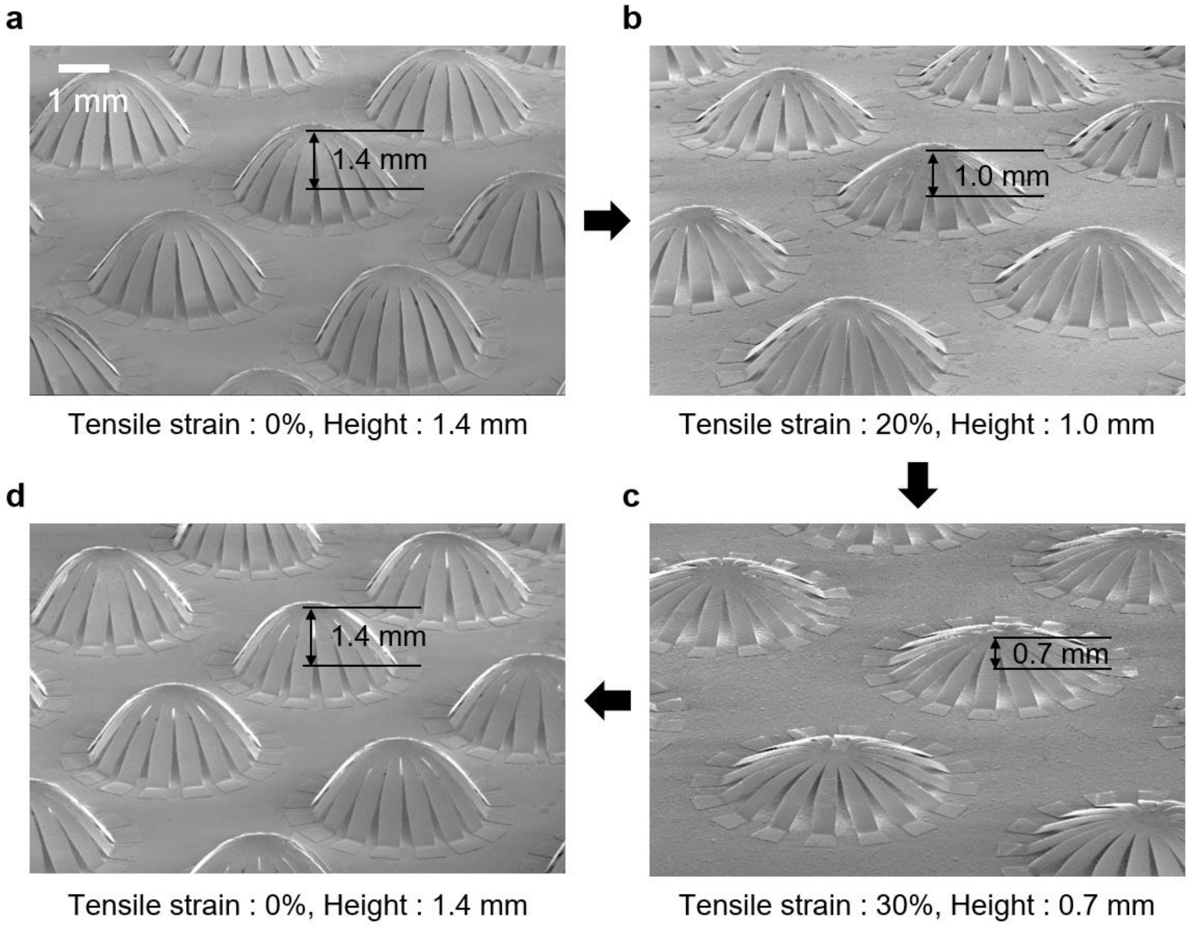

Supplementary Figure 14 | SEM mapping under in-plane stretching. SEM images of an array of 3D photodetector systems were taken under different in-plane biaxial stretching states (a-d). Each 3D structure in the 5×5 array stretches uniformly in the lateral direction through reductions in the height. The systems return to their original dimensions after release (same height before and after releasing the stretching; (**a** and **d**). No cracks or delaminations appear during repeated stretching, thereby confirming the excellent mechanical robustness of 3D pop-up hemispherical photodetectors.

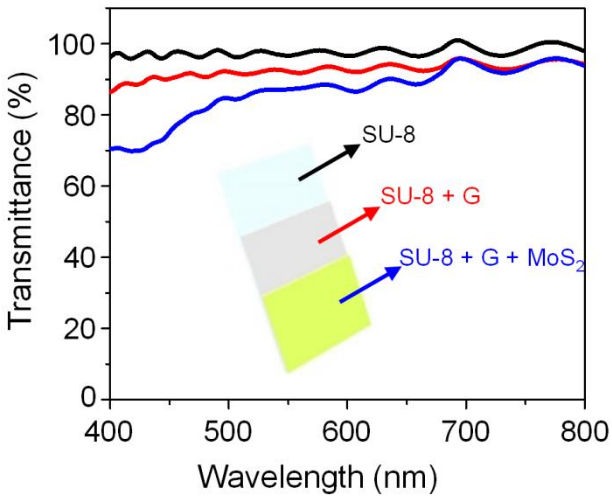

Supplementary Figure 15 | Estimation of transparency of different regions of 3D photodetector. Black line indicates the SU-8 film, red line indicates graphene electrode on SU-8 and blue line indicates MoS_2/Graphene/SU-8 structure.

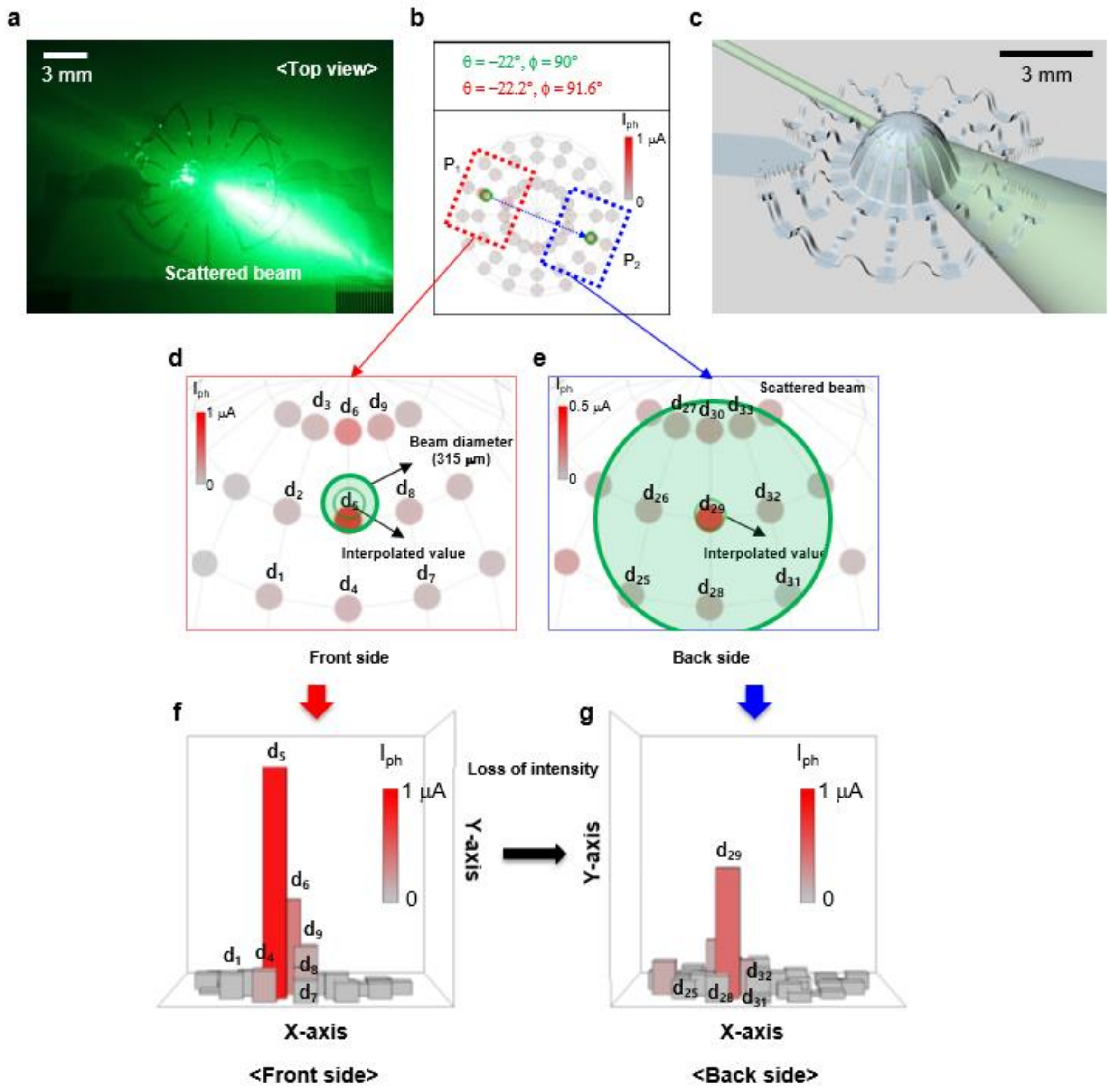

Supplementary Figure 16 | The role of scattered laser light in identifying the position and direction of the incident laser beam. a, Optical photograph, **b**, photoimaging results, and **c**, schematic illustrations of the intentional condition for maximum scattered light. Scattering affects most strongly the photoresistors near the location where the laser beam enters and exits the 3D surface. This feature is confirmed by the recorded photoresponse (**d** and **e**) and its intensity distribution (**f** and **g**) from nearest photoresistors that are not directly illuminated by the laser beam. Among 9 devices which were considered to identify the position and direction of the laser beam, only d_5 at P_1 and d_{29} at P_2 respond to the incident light while rest of the devices respond only due to scattered light. The photoresponse at P_2 (**g**) decreases as compared to P_1 (**f**) due to the loss of light intensity in passing through the 3D shape. These data highlight the importance of transparency.

a

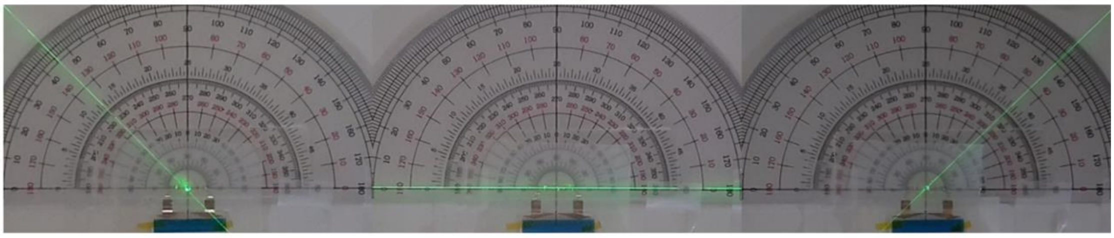

b

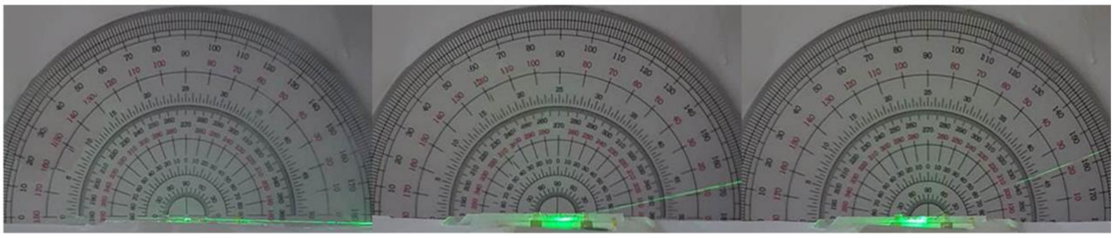

Supplementary Figure 17 | Angle measurement of incident laser on 3D photodetector. Adjustment of the laser incident angle a, with the range from -45° to 45° for θ and 90° for ϕ and **b**, with the range of incident angle from 90° to 67.5° for ϕ and 0° for θ to 3D photodetector with a protractor.

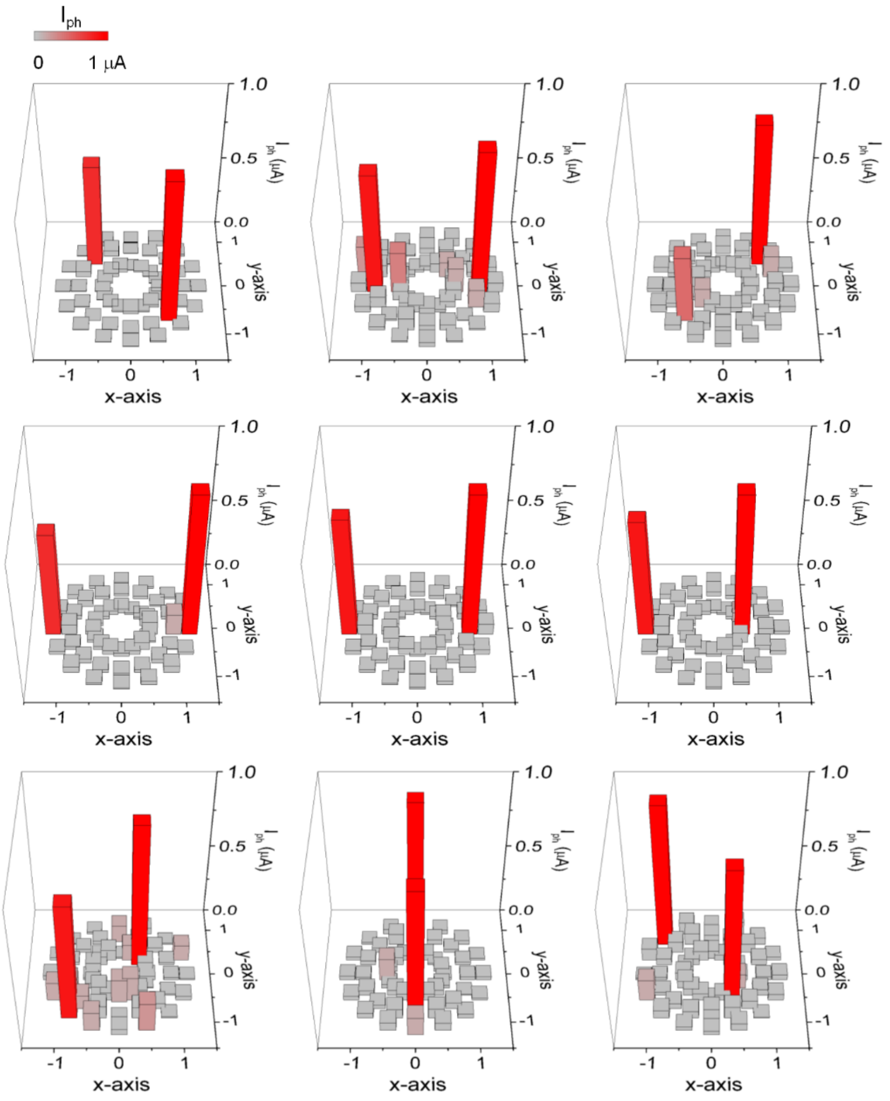

Supplementary Figure 18 | Photocurrent maps of 3D hemispherical photodetector. All the MoS_2 photodetectors expressed by bar graphs corresponding to the cases in Figure 4.

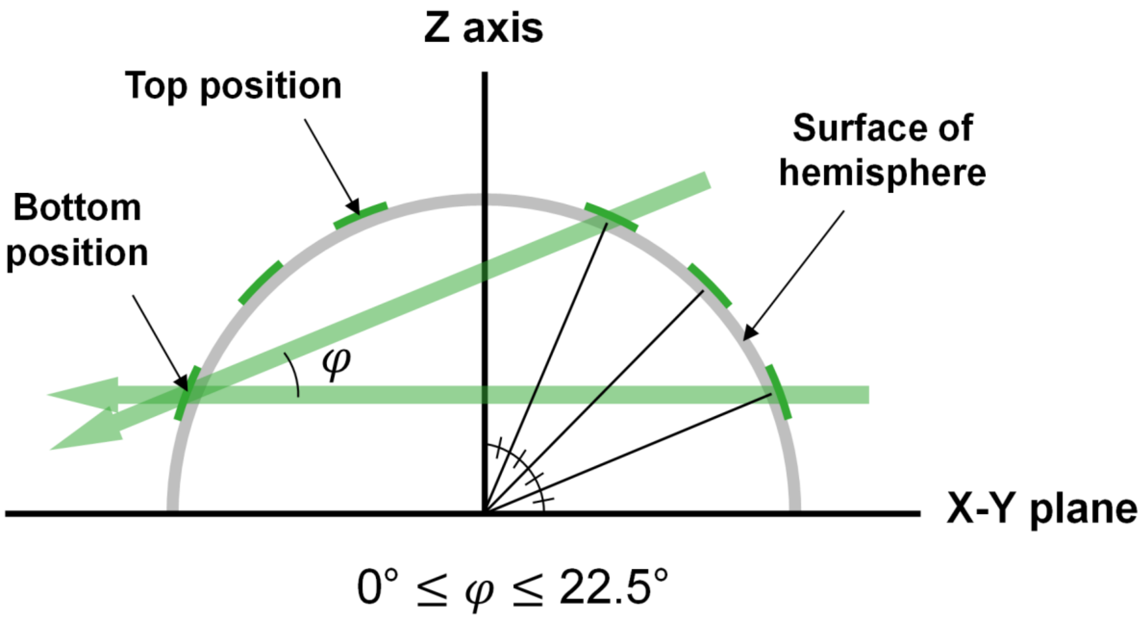

Supplementary Figure 19 | Schematic illustration of incident laser on 3D photodetector (low density array). The detectable range of φ is from 0° to 22.5°.

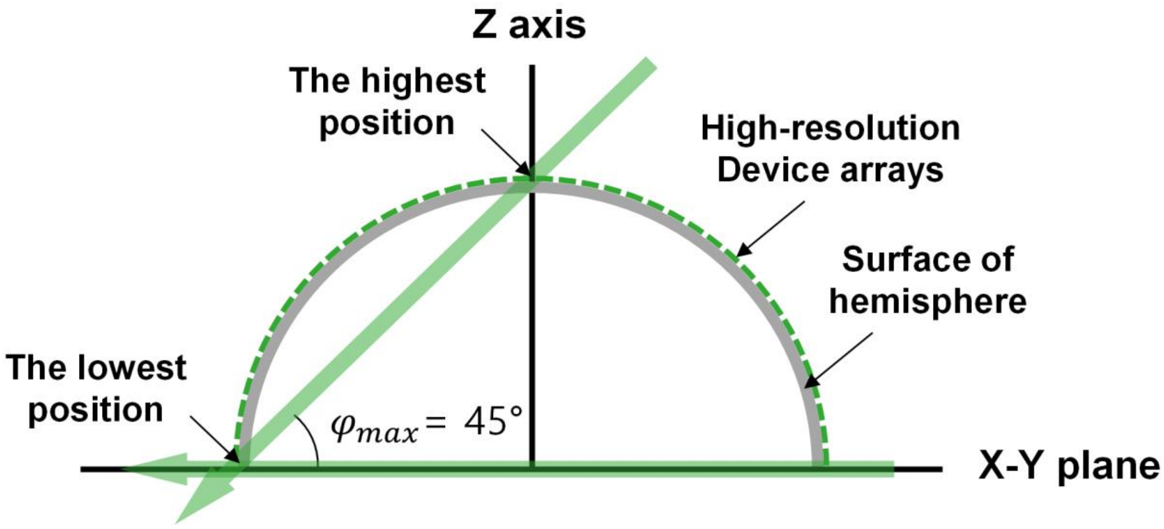

Supplementary Figure 20 | Schematic illustration of incident laser on 3D photodetector (high density array). The maximum detectable azimuth angle (φ_{max}) is 45°.

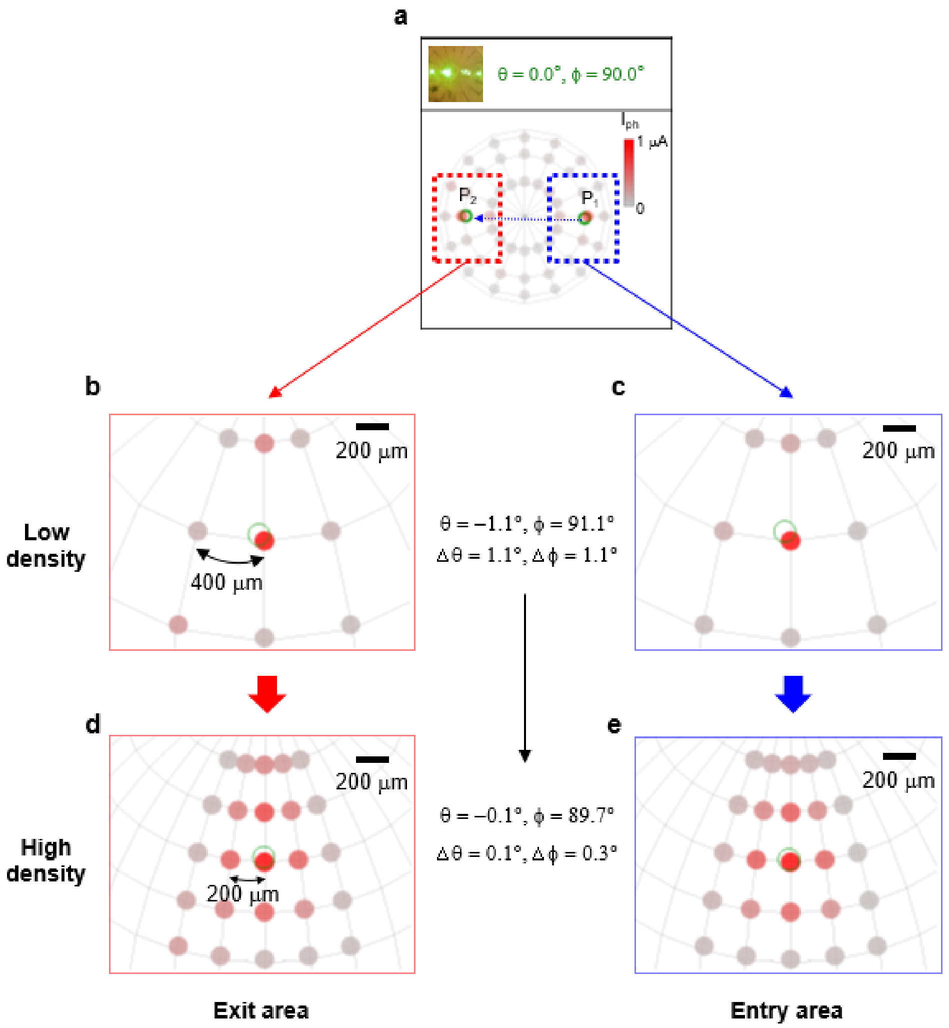

f

Reference angle	θ = −45.0° ϕ = 90.0°	θ = 0.0° ϕ = 90.0°	θ = 45.0° ϕ = 90.0°	θ = 0.0° ϕ = 90.0°	θ = 0.0° ϕ = 78.7°	θ = 0.0° ϕ = 67.5°	θ = 45.0°, ϕ = 67.5°	θ = 90.0° ϕ = 90.0°	θ = 135° ϕ = 112.5°	Average deviation
Low density	Δθ = 0.0° Δϕ = 0.3°	Δθ = 1.1° Δϕ = 1.1°	Δθ = 0.7° Δϕ = 1.3°	Δθ = 0.7° Δϕ = 0.5°	Δθ = 0.1° Δϕ = 1.4°	Δθ = 0.4° Δϕ = 1.3°	Δθ = 0.3° Δϕ = 1.7°	Δθ = 0.5° Δϕ = 0.4°	Δθ = 0.5° Δϕ = 1.4°	Δθ = 0.5° Δϕ = 1.0°
High density	Δθ = 0.1° Δϕ = 0.1°	Δθ = 0.1° Δϕ = 0.3°	Δθ = 0.1° Δϕ = 0.1°	Δθ = 0.2° Δϕ = 0.3°	Δθ = 0.1° Δϕ = 1.2°	Δθ = 0.1° Δϕ = 1.1°	Δθ = 0.1° Δϕ = 1.4°	Δθ = 0.1° Δϕ = 0.1°	Δθ = 0.1° Δϕ = 1.2°	Δθ = 0.1° Δϕ = 0.6°

Supplementary Figure 21 | Results that highlight the effects of distances among photoresistors in a 3D imaging system. A particular incident condition of laser beam is considered (**a**) and measurements for identifying the positions are performed for a low density array (**b** and **c**) and for a high density array (distances between photoresistors are half of those in the low density array) (**d** and **e**). The measurement results are tabulated in the form of deviation between measured and actual position of the beam. The measured deviation is less for the high density array as compared to the low density array of devices, indicating that the accuracy in identifying the position of the laser beam increases with decreasing the distances among fabricated devices.

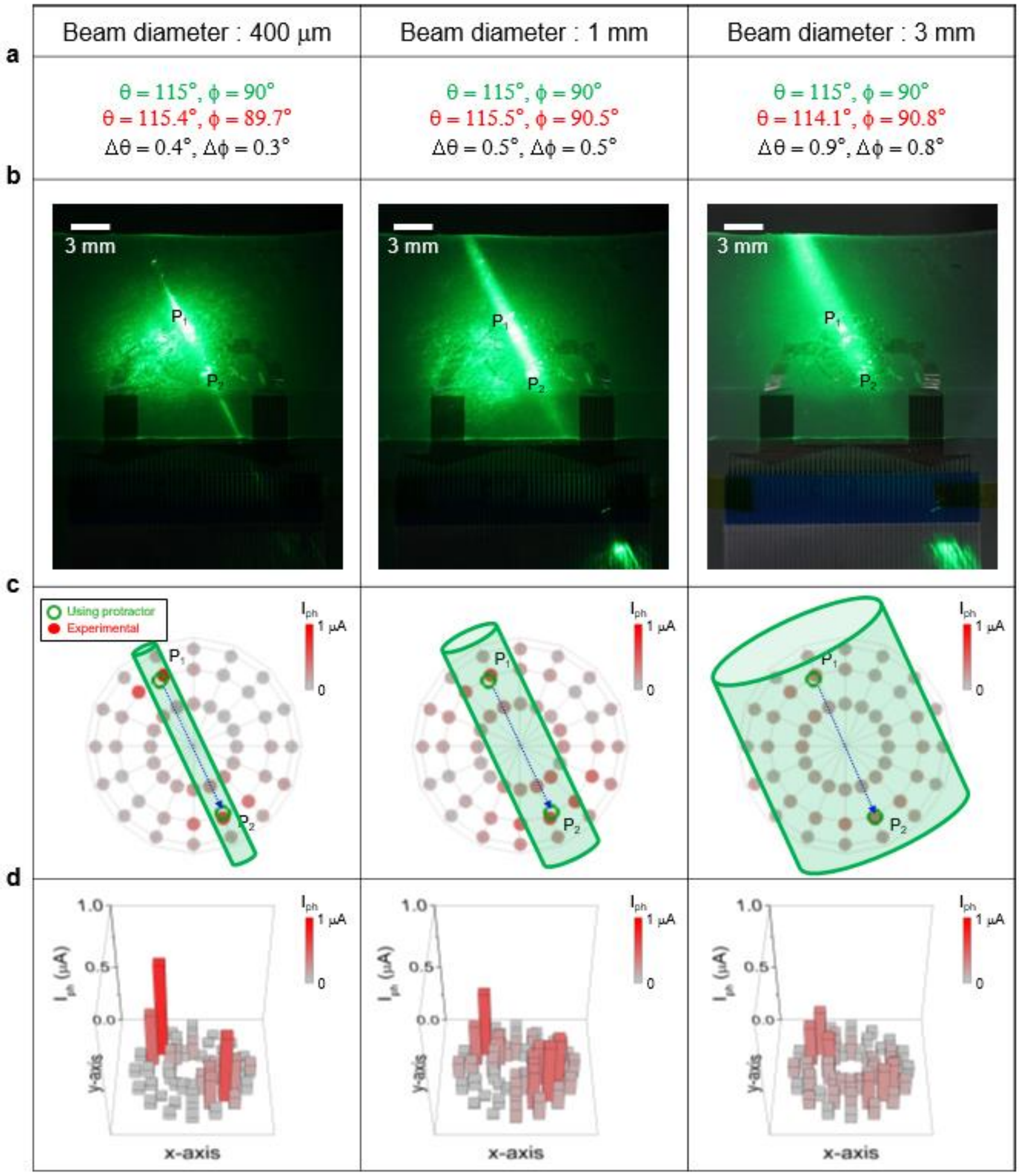

Supplementary Figure 22 | The effect of beam size on the accuracy of identifying the position of illumination. The incident point of laser beam on 3D hemispherical structure was identified (in the form of deviation, **a**) by changing the beam diameter (**b** and **c**) without changing the positions of the laser and 3D photodetector. The obtained photoresponses at P_1 and P_2 (**d**) for three different beam diameters (**b** and **c**) were used to calculate the deviation (**a**) between measured incident and actual position. The measured deviation was larger for larger beam diameters, indicating that the accuracy decreases with increasing laser spot size.

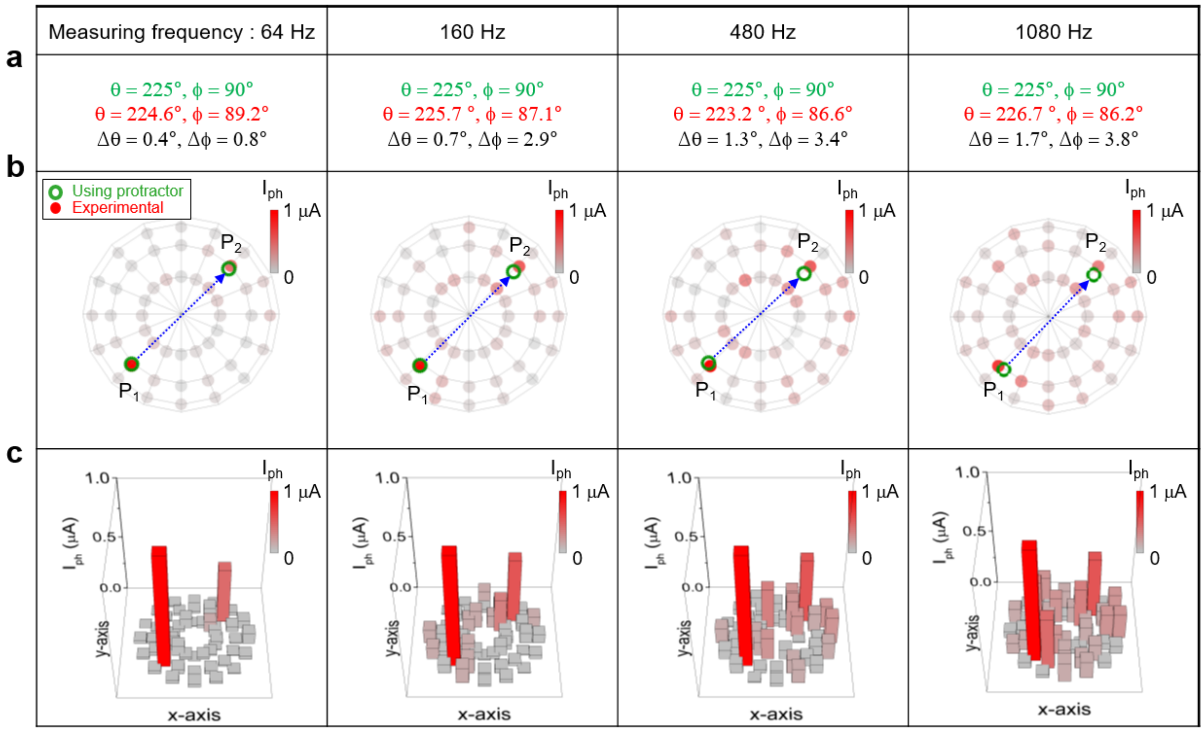

Supplementary Figure 23 | High frequency photoimaging capabilities with a 3D hemispherical photodetector. The positions of incident laser beam at entry (P_1) and exit (P_2) regions were identified (**b**) through the measured photoresponses (**c**) with increasing frequency. Although the deviation between measured position and actual one (**a**) slightly increases with frequencies, the system can identify the positions of the laser beam well into the kHz range (**b**, right).

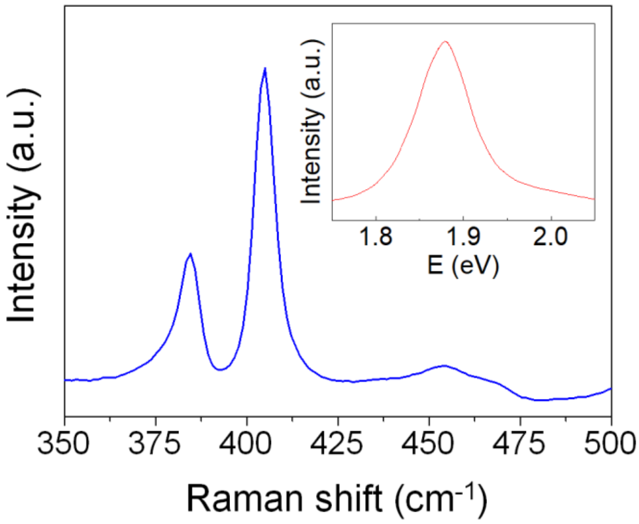

Supplementary Figure 24 | Raman Mapping of MoS_2. The raman spectrum of the used mono-layer MoS_2 grown by CVD method. Inset shows its room temperature photoluminescence spectrum.

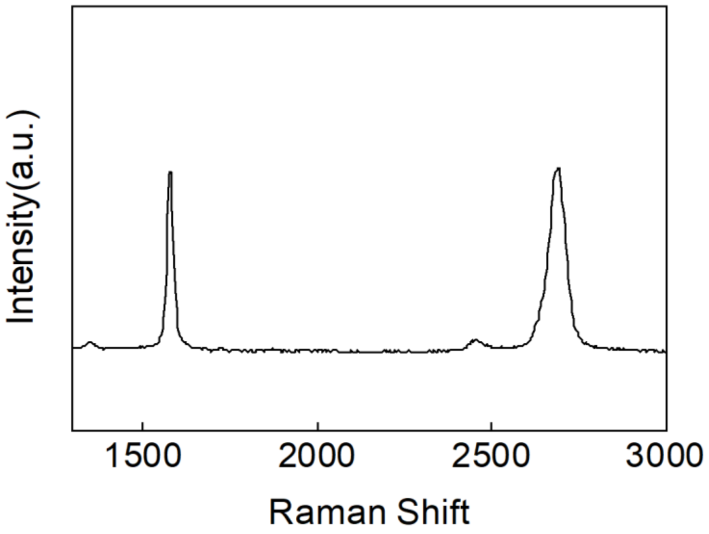

Supplementary Figure 25 | Raman mapping of graphene. The raman spectrum of double-layer graphene was recorded.

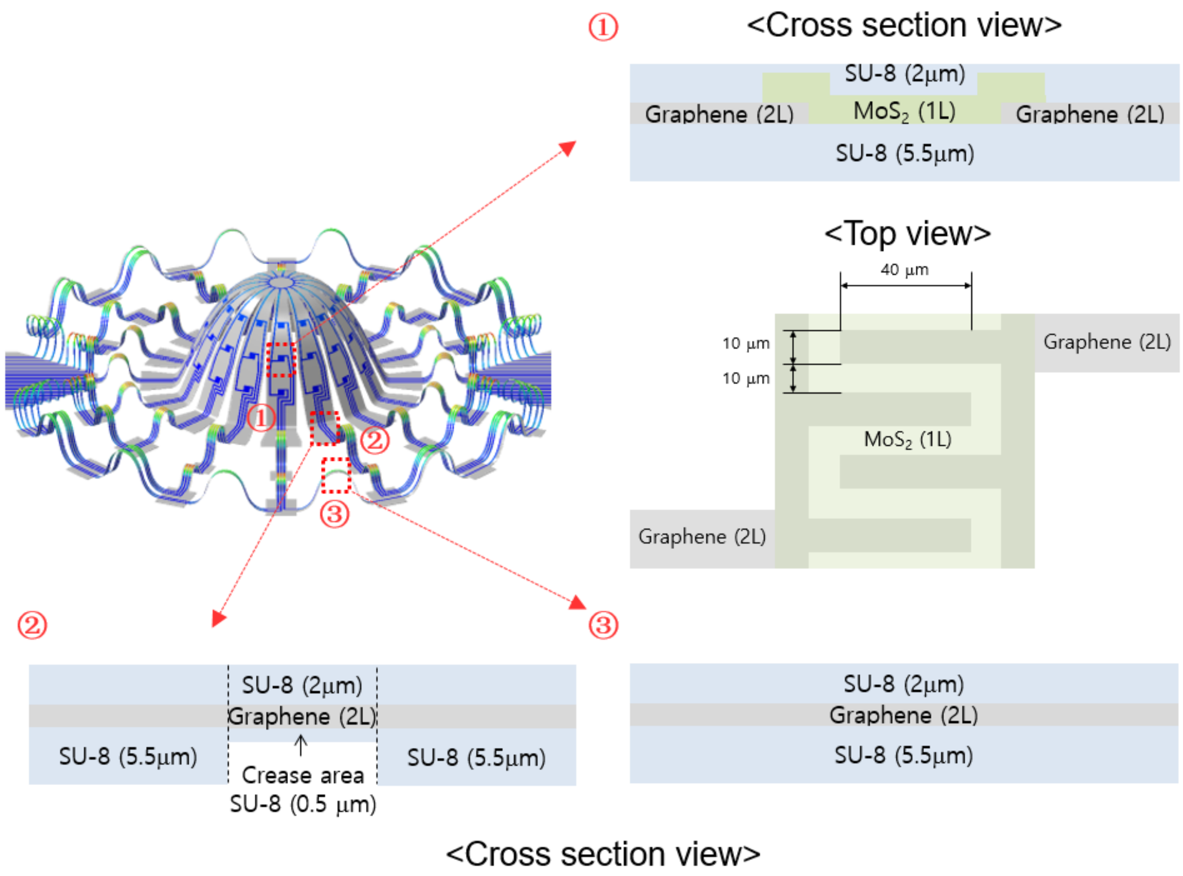

Supplementary Figure 26 | Detailed information on multilayer structures used in different regions of a 3D hemispherical photodetector structure: ①, The active part (cross section and top views) of the 3D structure includes monolayer MoS_2 as a photoactive layer with interdigitated bilayer graphene electrodes, all encapsulated between layers of SU-8. The channel length and width are 10 μm and 40 μm, respectively. ② and ③, Bilayer graphene passivated with SU-8 layers serve as interconnects and connections to the electrodes; the crease regions use thinner bottom SU-8 (②) while the other regions have relatively thicker bottom SU-8 (③).

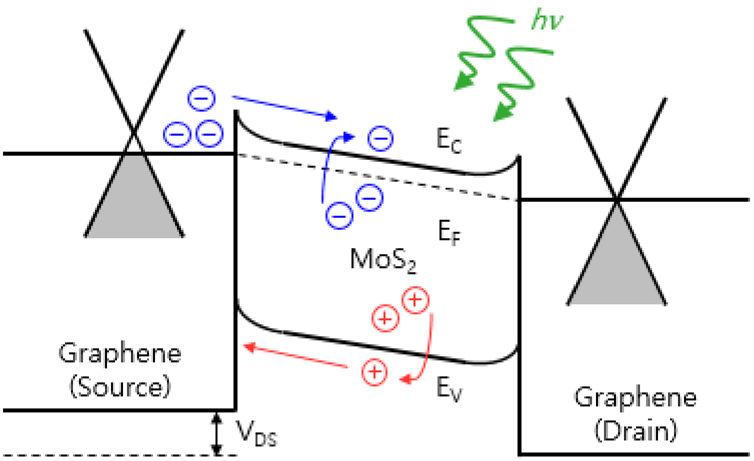

Supplementary Figure 27 | Band alignments at the graphene/MoS_2 interfaces during the photo-operation of a MoS_2 based resistive photodetector. Charge carriers, electrons (blue) and holes (red), are generated upon illumination and they are collected with the application of bias voltages, resulting in photocurrent. The solid lines indicate the valence band (E_V) and conduction band (E_C), while the dashed line represents the Fermi level. For $V_{DS} > 0$, the holes move toward the valence band (red arrow) and electrons move toward the conduction band (blue arrow).
