# Supplementary material for: Two-dimensional materials in functional three-dimensional architectures with applications in photodetection and imaging
Source: Nat Commun. 2018 Apr 12;9:1417. doi: 10.1038/s41467-018-03870-0 (PMC5897379; doi:10.1038/s41467-018-03870-0)
Supplement: Supplementary file 2 — Description of Additional Supplementary Files [file 41467_2018_3870_MOESM2_ESM.pdf]

Description of Additional Supplementary Files

File Name: Supplementary Movie 1
Description: FEA of the 3D hemisphere structures during compressive buckling, showing the distributions of maximum principal strains.

File Name: Supplementary Movie 2
Description: The stability of 3D photodetector array for stretching, bending and twisting.

File Name: Supplementary Movie 3
Description: In situ measurement of 3D photodetector.
